# Hydrogen Bonding and Polymorphism of Amino Alcohol Salts with Quinaldinate: Structural Study

**DOI:** 10.3390/molecules27030996

**Published:** 2022-02-01

**Authors:** Nina Podjed, Barbara Modec

**Affiliations:** Faculty of Chemistry and Chemical Technology, University of Ljubljana, Večna pot 113, 1000 Ljubljana, Slovenia; nina.podjed@fkkt.uni-lj.si

**Keywords:** hydrogen bond, synthon, crystal structure, polymorphism, amino alcohols, quinaldinic acid

## Abstract

Three amino alcohols, 3-amino-1-propanol (abbreviated as 3a1pOH), 2-amino-1-butanol (2a1bOH), and 2-amino-2-methyl-1-propanol (2a2m1pOH), were reacted with quinoline-2-carboxylic acid, known as quinaldinic acid. This combination yielded three salts, (3a1pOHH)quin (**1**, 3a1pOHH^+^ = protonated 3-amino-1-propanol, quin^−^ = anion of quinaldinic acid), (2a1bOHH)quin (**2**, 2a1bOHH^+^ = protonated 2-amino-1-butanol), and (2a2m1pOHH)quin (**3**, 2a2m1pOHH^+^ = protonated 2-amino-2-methyl-1-propanol). The 2-amino-1-butanol and 2-amino-2-methyl-1-propanol systems produced two polymorphs each, labeled **2a**/**2b** and **3a**/**3b**, respectively. The compounds were characterized by X-ray structure analysis on single-crystal. The crystal structures of all consisted of protonated amino alcohols with NH_3_^+^ moiety and quinaldinate anions with carboxylate moiety. The used amino alcohols contained one OH and one NH_2_ functional group, both prone to participate in hydrogen bonding. Therefore, similar connectivity patterns were expected. This proved to be true to some extent as all structures contained the NH_3_^+^∙∙∙^−^OOC heterosynthon. Nevertheless, different hydrogen bonding and *π*∙∙∙*π* stacking interactions were observed, leading to distinct connectivity motifs. The largest difference in hydrogen bonding occurred between polymorphs **3a** and **3b**, as they had only one heterosynton in common.

## 1. Introduction

Crystal engineering, defined as preparation of new molecular solids with tailor-made properties by using intermolecular interactions [1], continues to draw the interest of a wide scientific community. A rational design of these solids is based on a thorough understanding of the supramolecular chemistry of functional groups, in particular those with a hydrogen bonding potential. Owing to their strength and directionality, hydrogen bonds are likely to dominate above all the other interactions. The extensive surveys of the Cambridge Structural Database (CSD) helped with the formulation of empirical guidelines concerning the design of molecular crystals [2]. A generally valid rule on hydrogen bonding states that all good proton donors and acceptors are normally engaged in interactions [3]. A new terminology has also emerged: a pair of complementary functional groups, linked via intermolecular interaction, such as a hydrogen bond, is known as a synthon [4]. A heterosynthon is composed of two different functional groups, whereas two identical groups make part of a homosynthon. A prominent example of a self-association motif is a well-known carboxylic acid dimer. Another rule concerns the synthon hierarchy: the heterosynthons are favored over the homosynthons. Recent reports agree that it is still impossible to predict the structure of the molecular solid [5,6]. In this context, a phenomenon of polymorphism is brought up. The term polymorphism describes the existence of the same compound in several crystal forms that differ in spatial arrangements of their components and some of their properties [7]. Polymorphs of the same compound generally differ in lattice energies by a few kJ/mol at most [8]. As claimed by McCrone [9], the number of forms known for a given compound is proportional to the time and money spent in research on that compound. A systematic study of crystal structures of a large number of molecular solids, fueled also by the pharmaceutical industry [10,11], has revealed that at least every other molecule exhibits polymorphism [12]. It has been shown that hydrogen bonding potential only slightly increases a likelihood for the molecule to be polymorphic, whereas chiral molecules are somewhat reluctant towards crystallization in more than one crystal form [13].

**Figure 1:**
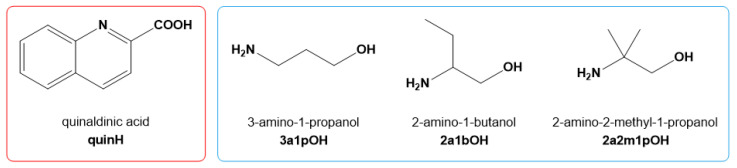
Structural formulae of quinaldinic acid, 3-amino-1-propanol, 2-amino-1-butanol, and 2-amino-2-methyl-1-propanol.

Herein, the solid-state structures of salts of three amino alcohols with quinaldinic acid are presented. The structural formulae of the acid and amino alcohols are depicted in Figure 1.

The salts contained protonated amino alcohols as cations and quinaldinate ions as counter-anions. Single crystals of all were obtained inadvertently as by-products of the [Cu(quin)_2_(H_2_O)] reactions with the amino alcohol [14]. It has been observed previously that the amino alcohol OH group undergoes a spontaneous deprotonation in the presence of copper(II) complexes [15]. The resulting amino alcoholate ions coordinated to copper(II) in a chelating manner with the alkoxide oxygen serving as a bridge between two or among three metal ions. The amino alcoholate coordination probably assists in the deprotonation of amino alcohol. Some of our reaction systems provided a few more pieces of information concerning the formation of the amino alcoholate ions. The nature of the products, isolated from these reaction systems, strongly suggests a proton transfer from the OH group of the amino alcohol molecule to the NH_2_ group of another molecule. In the reaction below, the H_2_N–(CH_2_)*_n_*–OH denotes amino alcohol in general.

2 H_2_N–(CH_2_)*_n_*–OH ↔ H_3_N^+^–(CH_2_)*_n_*–OH + H_2_N–(CH_2_)*_n_*–O^−^

The H_2_N–(CH_2_)*_n_*–O^−^ ions coordinated to copper(II), whereas the H_3_N^+^–(CH_2_)*_n_*–OH ions crystallized as salts with quinaldinate. Later, a more straightforward synthesis of these salts was sought. A reaction of quinaldinic acid with the excess of amino alcohol in methanol with no copper(II) complex involved was met with success. Two of the salts were found to be polymorphic. A detailed account of the solid-state structures follows.

## 2. Results and Discussion

First, the common structural features of the title compounds are described. The crystal structures of all consist of NH_2_-protonated amino alcohol molecules as counter-cations and quinaldinate anions with carboxylate moiety. In all, the C–O bond lengths of the carboxylate are the same within the experimental error. Interestingly, in some structures, the quinaldinate ions deviate from planarity. For convenience, we have described this deviation as a twist angle between the carboxylate plane and the quinoline plane. Depending upon the structure, the quinaldinates can stack one upon another. Geometric parameters of the *π*∙∙∙*π* stacking interactions are conventionally given by the centroid∙∙∙centroid distance, dihedral angle, and shift distance [16]. Quinaldinate can participate in another interaction, a C–H∙∙∙*π* interaction. All interactions involving *π* rings are given in Table 1. Both the cations and the anions possess groups that are hydrogen bond donors (NH_3_^+^ in protonated amino alcohol) or acceptors (carboxylate and quinaldinate nitrogen) or both (OH in protonated amino alcohol). With the first two being good hydrogen bond donors/acceptors, their participation in hydrogen bonding is likely to govern the connectivity patterns in solid state. A detailed list of hydrogen bonds is given in Table 2, whereas all possible heterosynthons and their actual occurrences in the structures of the title compounds are given in Table 3.

**Figure 2:**
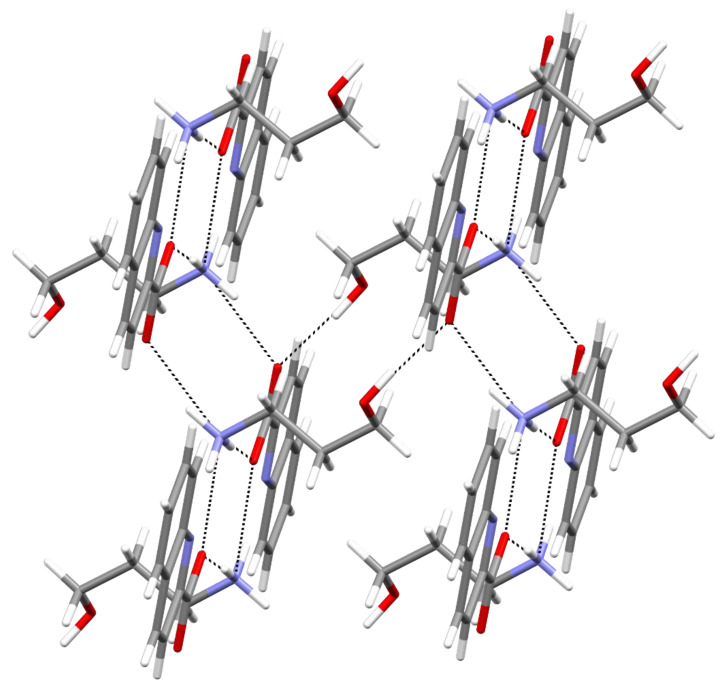
Perpendicular view to the section of a layer of hydrogen-bonded cations and anions in the structure of **1**.

The crystal structure of **1** consists of 3a1pOHH^+^ cations and strictly planar quinaldinate ions. All hydrogen bond donors and acceptors participate in intermolecular interactions. The quinaldinate nitrogen interacts only weakly with the NH_3_^+^ group: the corresponding N∙∙∙N distance amounts to 3.108(3) Å, the value that is almost the same as the sum of the van der Waals radii for nitrogen atoms, 3.1 Å [17]. The connectivity pattern consists of two types of hydrogen bonds: the OH∙∙∙^−^OOC and the NH_3_^+^∙∙∙^−^OOC hydrogen bonds. Each type occurs between the cation and the anion. The hydrogen bonding pattern produces infinite layers, which are coplanar with the *ab* plane and stack along the *c* crystallographic axis. Section of such a layer is depicted in Figure 2. The layers stack upon one another with significant *π*∙∙∙*π* stacking interactions occurring between quinaldinates from adjacent layers (Figure S4). Parameters of the shortest *π*∙∙∙*π* stacking interaction are Ph∙∙∙Py type, *Cg*∙∙∙*Cg* = 3.6571(15) Å, dihedral angle = 0.41(11)°, shift distance = 1.346 Å.

**Figure 3:**
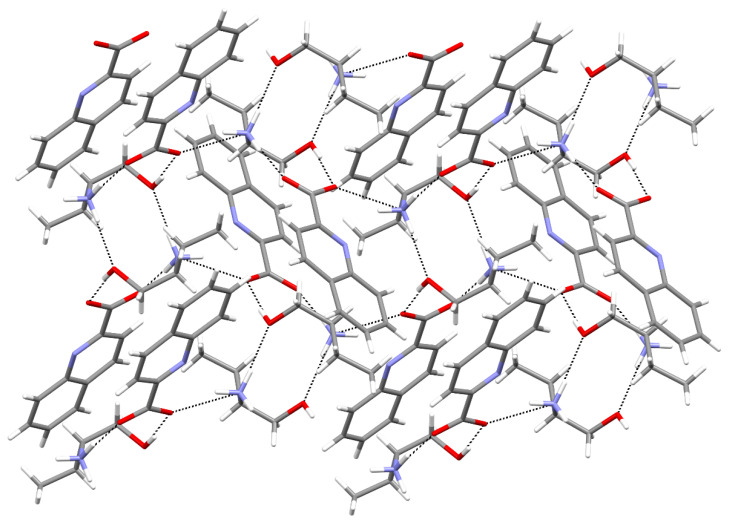
A perpendicular view to the layer in **2a**.

**Figure 4:**
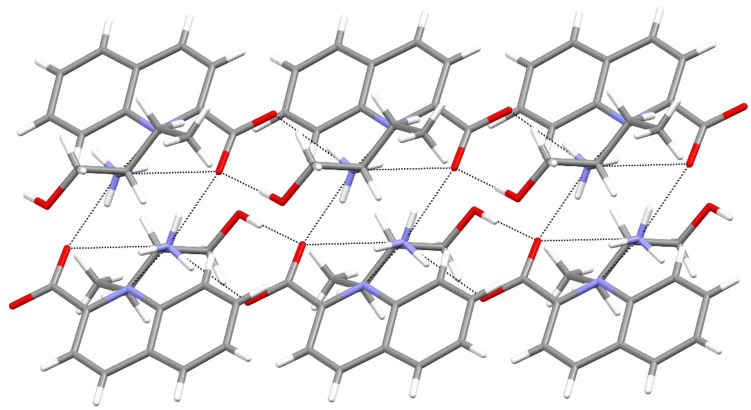
Section of a chain in **2b**.

The 2-amino-1-butanol salt was found in two polymorphic forms, **2a** and **2b**. Both crystallize in a monoclinic *P* 2_1_/*n* unit cell. The quinaldinates of **2a** are non-planar with the twist angle of 11.4(2)°, whereas those of **2b** are nearly planar. The structures of both feature the OH∙∙∙^−^OOC and the NH_3_^+^∙∙∙^−^OOC synthons. In **2a**, a weak interaction occurs between NH_3_^+^ and OH groups. Once again, in neither of the two structures, the quinaldinate nitrogen is engaged in stronger intermolecular interactions. Its shortest contact occurs with the NH_3_^+^ group with the corresponding N∙∙∙N distance being 3.1669(16) Å (**2a**) or 3.080(3) Å (**2b**). Hydrogen bonds link cations and anions into layers (polymorph **2a**, Figure 3) or into chains (polymorph **2b**, Figure 4). In **2a**, significant *π*∙∙∙*π* stacking interactions occur between quinaldinates from adjacent layers (Figure S5). Parameters of the shortest *π*∙∙∙*π* stacking interaction are Ph∙∙∙Py type, dihedral angle = 0.39(7)°, *Cg*∙∙∙*Cg* = 3.5163(9) Å, and shift distance = 1.123 Å. The packing of chains in **2b** is such that no *π*∙∙∙*π* stacking occurs.

**Figure 5:**
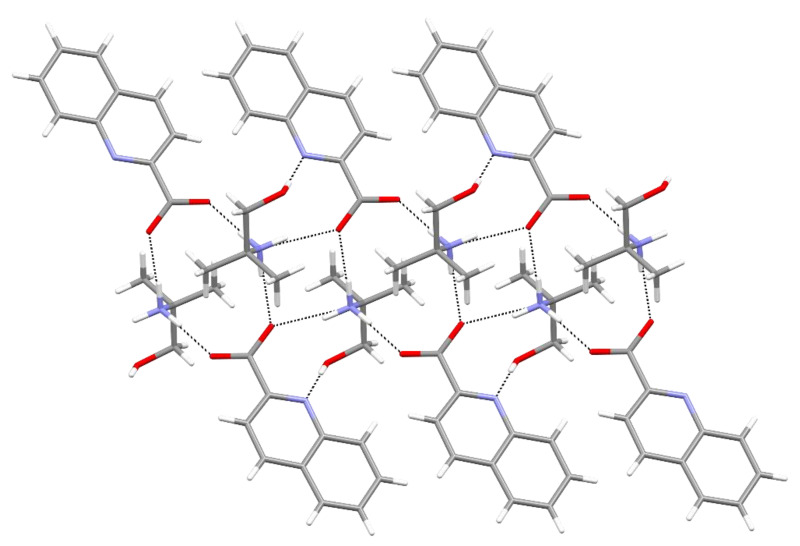
Section of a chain in **3a**.

The 2-amino-2-methyl-1-propanol salt also exists in two polymorphic forms. The one that crystallizes in a monoclinic *P* 2_1_/*n* cell was labeled **3a**, and the one that crystallizes in a triclinic *P*−1 cell was labeled **3b**. The quinaldinates of the **3a** polymorph are non-planar with the twist angle of 25.48(10)°. Apart from the usual synthon, the NH_3_^+^∙∙∙^−^OOC hydrogen bond, there is a short contact between the hydroxyl group of the 2a2m1pOHH^+^ cation and the quinaldinate nitrogen with the O∙∙∙N distance being 2.8187(17) Å. The NH_3_^+^∙∙∙^−^OOC and the OH∙∙∙N(quin^−^) hydrogen bonds link ions into chains, which propagate along *a* crystallographic axis (Figure 5). The chains pack in a parallel fashion without any *π*∙∙∙*π* stacking interactions.

**Figure 6:**
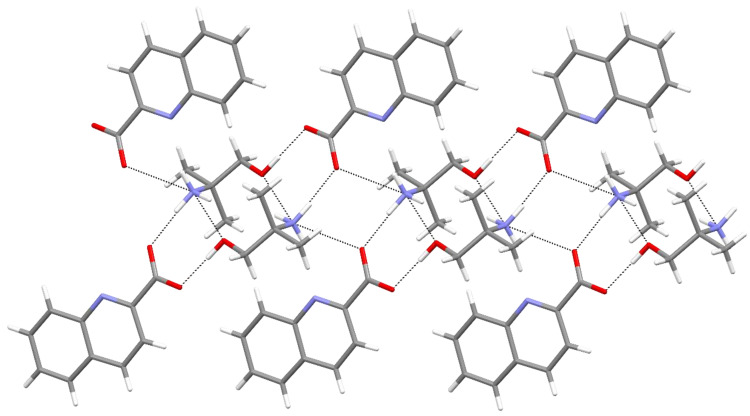
Section of a chain in **3b**.

The **3b** polymorph also consists of infinite chains. The chains propagate along *b* crystallographic axis. Yet, the hydrogen bonding motif markedly differs from that in **3a**. Firstly, the quinaldinate nitrogen is engaged in weak interaction with the adjacent NH_3_^+^ moiety. The corresponding N∙∙∙N contact is 3.1037(14) Å. In the infinite chain, the following synthons may be recognized: in addition to the usual NH_3_^+^∙∙∙^−^OOC and OH∙∙∙^−^OOC hydrogen bonds, there is also the NH_3_^+^∙∙∙OH hydrogen bond that links the cations (Figure 6). Of the two polymorphs, only **3b** displays hydrogen-bonding interactions between the cations. The packing of the chains is such that it allows *π*∙∙∙*π* stacking interactions between neighboring chains (Figure S6). The quinaldinates are again non-planar with the 17.26(9)° twist angle.

Products obtained upon a direct reaction of a specific amino alcohol and quinaldinic acid may be classified as salts. The combinations involving amines and carboxylic acids do not always produce salts. The frequently employed *∆*pK_a_ rule in predicting the nature of the product [18], ionic (a salt) or neutral (a co-crystal), can give indefinite answers. It has been stated that with the difference between the pK_a_ of the base and the pK_a_ of the acid in the −1 to 4 interval, the ionization of functional groups depends upon the whole crystal packing [18], and the product classification depends upon the position of the proton along a N∙∙∙O hydrogen bond [19]. The combinations of amino alcohols, used in place of amines, and quinaldinic acid (quinoline-2-carboxylic acid) result in the *∆*pK_a_ values that do not fall into the −1 to 4 domain. Although the hydroxyl group lowers the pK_a_ value relative to the “parent” alkylamine (For example, pK_a_ of 2-aminoethanol is by 1.15 unit lower than pK_a_ of ethylamine, 9.50 vs. 10.65 [20]), it is the quinaldinic acid that swings the balance in favor of the salt formation. The salt formation was further confirmed for all title compounds in the process of structure refinement by the location of proton in the electron difference maps.

The components of the three salts contain the same functional groups. Similar connectivity patterns are thus expected. The following discussion shows to what extent this expectation was realized. It is to be noted that three compounds present a very limited data set. The general validity of the conclusions is thus to be treated with caution. Firstly, in the solid-state structures of title compounds, all good proton donors and acceptors are used in the intermolecular connectivity. All five structures conform to the predicted synthon hierarchy [2]: only the heterosynthons may be displayed and no homomeric ones. As shown in Table 3, all our salts feature the NH_3_^+^∙∙∙^−^OOC synthon. The second one in the order of occurrence is the OH∙∙∙^−^OOC synthon, which is observed in all but **3a**. Interestingly, its formation is with no exception accompanied by a weak NH_3_^+^∙∙∙N(quin^−^) interaction. The **3a** salt, which lacks the OH∙∙∙^−^OOC interaction, also lacks the NH_3_^+^∙∙∙N(quin^−^) interaction. The absence of the NH_3_^+^∙∙∙N(quin^−^) interaction in **3a** is compensated by the OH∙∙∙N(quin^−^) hydrogen bond. The salt **3a** is the only compound that demonstrates this type of hydrogen bond; **3b**, the other (2a2m1pOHH)quin polymorph, also displays a specific feature, a NH_3_^+^∙∙∙OH interaction. The latter is of interest because it occurs between ions of the same type, i.e., the 2a2m1pOHH^+^ cations. The survey reveals that **1** and **2b** feature the same heterosynthons. The same observation pertains to the **2a**/**3b** pair. The **3a** polymorph differs from the other four structures. According to the literature, each pair of polymorphs, the **2a**/**2b** polymorphs and the **3a**/**3b** polymorphs, with differences in hydrogen bonding between their components may be thus classified as hydrogen bond isomers of the same solid [21]. The **2a**/**2b** polymorphs crystallized from the same reaction mixture, as opposed to the **3a**/**3b** polymorphs, which crystallized from different reaction mixtures. The **2a**/**2b** polymorphs are therefore concomitant polymorphs [22]. The structures of **2a** and **2b** reveal another important difference. Whereas **2a** features *π*∙∙∙*π* stacking of quinaldinates, this type of interaction is lacking in **2b**. The same difference pertains to the **3a**/**3b** pair. On the other hand, the structures of all four share a common feature: the C–H∙∙∙*π* type interactions.

The structures of **1**–**3b** have some structural features in common. The observed differences are a result of a complex interplay of short- and long-range intermolecular interactions that govern the supramolecular assembly during the crystallization procedure. Yet, each structure thus presents a specific situation and as such conforms with the current opinion in the field of crystal engineering that it is impossible to predict all molecular recognition events during the crystallization.

## 3. Materials and Methods

**General.** All reagents but acetonitrile were obtained from commercial sources (Aldrich and Fluorochem) and used as received. Acetonitrile was dried over molecular sieves [23]. In the case of the 2-amino-1-butanol reagent, a racemic mixture was used. The copper starting material, [Cu(quin)_2_(H_2_O)], was synthesized as previously reported [24]. Infrared (IR) spectra were recorded with the ATR module in the 4000–400 cm^−1^ spectral range on a Bruker Alpha II FT-IR spectrophotometer (Bruker, Manhattan, MA, USA). No corrections were made to the spectra. The spectra of all reveal strong bands in the 1560–1520 and 1370–1360 cm^−1^ spectral regions, which may be assigned as the ν_as_(COO^−^) and ν_s_(COO^−^) absorptions of the ionized quinaldinate. The engagement of the OH and NH_3_^+^ functional groups in hydrogen bonding prevents unambiguous identification of the stretching/deformation bands of these functional groups. ^1^H nuclear magnetic resonance (NMR) spectra were recorded at 500 MHz on a Bruker Avance III 500 (Bruker BioSpin GmbH, Rheinstetten, Germany). The solvent was (CD_3_)_2_SO (DMSO-*d*_6_) containing 0.03% tetramethylsilane (TMS), and all spectra were referenced to the central peak of the residual resonance for DMSO-*d_6_* at 2.50 ppm [25]. ^1^H NMR spectra were processed using the MestReNova program [26]. Chemical shifts (δ) are given in ppm and coupling constants (*J*) in Hz. Multiplicities are labeled as follows: s = singlet, d = doublet, t = triplet, dd = doublet of doublet, and m = multiplet. Elemental analysis CHN was performed on a Perkin-Elmer 2400 II analyzer. Powder X-ray diffraction (PXRD) patterns were collected on a PANanlytical X’Pert PRO MD diffractometer (PANALYTICAL, Almelo, The Netherlands) using monochromatised Cu-K*_α_* radiation (*λ* = 1.5406 Å). Thermogravimetric analyses were performed on a Mettler Toledo TG/DSC 1 instrument (Mettler Toledo, Schwerzenbach, Switzerland). Samples were placed into a 150 μL platinum crucible. Initial masses of samples were around 10 mg. Samples were heated from 25 to 450 °C with a heating rate of 10 °C min^−1^ and the furnace was purged with air at a flow rate of 50 mL min^−1^. The baseline was subtracted. All three salts are stable up to about 120 °C and then the decomposition processes take place. No phase transitions were observed in the 25–120 °C temperature range.

**(3a1pOHH)quin (1)**. Quinaldinic acid (100 mg, 0.58 mmol), methanol (10 mL), and 3-amino-1-propanol (88 μL) were added to an Erlenmeyer flask. The mixture was stirred until all the solid was consumed. The resulting solution was left to stand at ambient conditions. On the following day, it was concentrated under reduced pressure on a rotary evaporator. A glass vial with diethyl ether was carefully inserted into the Erlenmeyer flask with the concentrate. Colorless crystals of (3a1pOHH)quin were filtered off. Yield: 106 mg, 74%. Notes. The identity of the product was confirmed by PXRD (Figure S1). Single crystals of **1** were obtained as follows. A Teflon container was filled with CuO (50 mg, 0.63 mmol), quinaldinic acid (120 mg, 0.69 mmol), acetonitrile (7.5 mL), and 3-amino-1-propanol (150 mg). The container was closed and inserted into a steel autoclave, which was heated for 24 h at 105 °C. Afterwards, the reaction mixture was allowed to cool slowly to room temperature. Black solid was filtered off, and the resulting green filtrate was concentrated under reduced pressure on a rotary evaporator. The concentrate was stored at 4 °C. A mixture of colorless crystals of (3a1pOHH)quin (**1**) and blue needle-like crystals of *trans*-[Cu(quin)_2_(3a1pOH)_2_] was obtained. ^1^H NMR (500 MHz, DMSO-*d*_6_ with 0.03% *v/v* TMS): δ 8.30 (1H, d, *J* = 8.4 Hz, quin^−^), 8.12 (1H, d, *J* = 8.4 Hz, quin^−^), 8.04 (1H, d, *J* = 8.4 Hz, quin^−^), 7.95 (1H, dd, *J* = 8.2, 1.1 Hz, quin^−^), 7.74–7.71 (1H, m, quin^−^), 7.60–7.57 (1H, m, quin^−^), 3.50 (2H, t, *J* = 6.0 Hz, 3a1pOHH^+^), 2.95 (2H, t, *J* = 7.3 Hz, 3a1pOHH^+^), 1.80–1.74 (2H, m, 3a1pOHH^+^) ppm. Elemental analysis calcd. for C_13_H_16_N_2_O_3_ (%): C, 62.89; H, 6.50; N, 11.28. Found (%): C, 62.80; H, 6.38; N, 11.35. IR (ATR, cm^−1^): 3352m, 3061m, 2989m, 2947m, 2888m, 2745m, 2503w, 2090w, 1593s, 1559s, 1519s, 1502s, 1475m, 1462s, 1425s, 1384s, 1372vs, 1335s, 1298m, 1276m, 1214m, 1184m, 1168s, 1145w, 1129w, 1104m, 1068s, 1042m, 1023m, 1001m, 950w, 903s, 891s, 878m, 849m, 790vs, 776vvs, 746s, 629s, 592s, 541w, 530m, 520s, 500w, 477m.

**(2a1bOHH)quin (2)**. Quinaldinic acid (100 mg, 0.58 mmol), methanol (10 mL), and 2-amino-1-butanol (109 μL) were added to an Erlenmeyer flask. The mixture was stirred until all the solid was consumed. The resulting solution was left to stand at ambient conditions. On the following day, it was concentrated under reduced pressure on a rotary evaporator. A glass vial with diethyl ether was carefully inserted into the Erlenmeyer flask with the concentrate. Colorless, needle-like crystals of (2a1bOHH)quin were filtered off. Yield: 116 mg, 77%. Notes. PXRD confirmed that the product is mostly **2b** polymorph (Figure S2). Single crystals of **2a** and **2b** polymorphs were obtained as follows. [Cu(quin)_2_(H_2_O)] (50 mg, 0.12 mmol), nitromethane (7.5 mL) and 2-amino-1-butanol (0.25 mL) were added to an Erlenmeyer flask. The mixture was stirred thoroughly until all the solid was consumed. After a few days, a mixture of crystals of **2a** and **2b** polymorphs was obtained. ^1^H NMR (500 MHz, DMSO-*d*_6_ with 0.03% *v/v* TMS): δ 8.30 (1H, d, *J* = 8.4 Hz, quin^−^), 8.08 (1H, d, *J* = 8.5 Hz, quin^−^), 8.00 (1H, d, *J* = 8.4 Hz, quin^−^), 7.95 (1H, dd, *J* = 8.1, 1.4 Hz, quin^−^), 7.76–7.72 (1H, m, quin^−^), 7.60–7.57 (1H, m, quin^−^), 3.64 (1H, dd, *J* = 11.7, 3.8 Hz, 2a1bOHH^+^), 3.50 (1H, dd, *J* = 11.7, 6.2 Hz, 2a1bOHH^+^), 3.04–2.99 (1H, m, 2a1bOHH^+^), 1.62–1.53 (2H, m, 2a1bOHH^+^), 0.91 (3H, t, *J* = 7.5 Hz, 2a1bOHH^+^) ppm. Elemental analysis calcd. for C_14_H_18_N_2_O_3_ (%): C, 64.11; H, 6.92; N, 10.68. Found (%): C, 64.06; H, 6.68; N, 10.77. IR of **2a** polymorph (ATR, cm^−1^): 3017w, 2965m, 2935m, 2873m, 2752m, 2635m, 2072w, 1594s, 1576s, 1554s, 1501s, 1462s, 1428m, 1371vvs, 1346s, 1306m, 1288w, 1272w, 1254m, 1219w, 1205m, 1171s, 1151m, 1133m, 1111w, 1066s, 1041s, 988s, 967w, 953m, 890m, 861s, 810s, 782vvs, 761s, 747s, 661m, 626s, 592s, 547w, 526w, 499m, 478m, 469m, 439m. IR of **2b** polymorph (ATR, cm^−1^): 3232w, 3063m, 2963m, 2936m, 2868m, 1590m, 1553s, 1519s, 1503s, 1459s, 1427m, 1388s, 1367vs, 1340s, 1253w, 1219w, 1205m, 1170m, 1148m, 1068s, 1011w, 973w, 954w, 917w, 892m, 863s, 811s, 787vvs, 753m, 689m, 627s, 596s, 543w, 520m, 506m, 479w, 455w.

**(2a2m1pOHH)quin (3)**. Quinaldinic acid (100 mg, 0.58 mmol), methanol (10 mL), and 2-amino-2-methyl-1-propanol (108 μL) were added to an Erlenmeyer flask. The mixture was stirred until all the solid was consumed. The resulting solution was left to stand at ambient conditions. On the following day, it was concentrated under reduced pressure on a rotary evaporator. A glass vial with diethyl ether was carefully inserted into the Erlenmeyer flask with the concentrate. Colorless crystals of (2a2m1pOHH)quin were filtered off. Yield: 111 mg, 73%. Notes. PXRD confirmed that the product is mostly **3b** polymorph (Figure S3). Single crystals of **3a** polymorph were obtained as follows. [Cu(quin)_2_(H_2_O)] (50 mg, 0.12 mmol), acetonitrile (7.5 mL), and 2-amino-2-methyl-1-propanol (0.5 mL) were added to an Erlenmeyer flask. The mixture was stirred thoroughly until all the solid was consumed. The resulting blue solution was left to stand at ambient conditions. On the following day, a mixture of colorless, needle-like crystals of **3a** polymorph and blue crystalline solid *syn*-[Cu_2_(quin)_2_(2a2m1pO)_2_] was obtained. Single crystals of **3b** polymorph were obtained as follows. Teflon container was filled with [Cu(quin)_2_(H_2_O)] (50 mg, 0.12 mmol), acetonitrile (7.5 mL) and 2-amino-2-methyl-1-propanol (0.5 mL). The container was closed and inserted into a steel autoclave, which was heated for 24 h at 105 °C. Afterwards, the reaction mixture was allowed to cool slowly to room temperature. The resulting blue solution was left to stand at ambient conditions. After a few days, a mixture of colorless, needle-like crystals of **3b** polymorph and blue crystalline solid *syn*-[Cu_2_(quin)_2_(2a2m1pO)_2_] was obtained. ^1^H NMR (500 MHz, DMSO-*d*_6_ with 0.03% *v/v* TMS): δ 8.30 (1H, d, *J* = 8.4 Hz, quin^−^), 8.10 (1H, d, *J* = 8.5 Hz, quin^−^), 8.01 (1H, d, *J* = 8.4 Hz, quin^−^), 7.94 (1H, dd, *J* = 8.1, 1.4 Hz, quin^−^), 7.75–7.72 (1H, m, quin^−^), 7.60–7.57 (1H, m, quin^−^), 3.43 (s, 2H, 2a2m1pOHH^+^), 1.22 (s, 6H, 2a2m1pOHH^+^) ppm. Elemental analysis calcd. for C_14_H_18_N_2_O_3_ (%): C, 64.11; H, 6.92; N, 10.68. Found (%): C, 63.97; H, 6.64; N, 10.71. IR of **3a** polymorph (ATR, cm^−1^): 3185w, 2980m, 2894m, 2829s, 2724m, 2633m, 2593m, 2543m, 2168w, 1630s, 1578s, 1549vs, 1503m, 1482m, 1467s, 1426m, 1385vs, 1372vs, 1345s, 1327m, 1299m, 1264m, 1213w, 1173s, 1148m, 1114m, 1095m, 1067vs, 1009w, 980w, 958w, 946w, 912w, 893m, 873m, 853m, 804s, 778vvs, 752s, 737s, 697m, 651m, 630s, 592s, 551m, 523m, 480m, 459vvs, 421m. IR of **3b** polymorph (ATR, cm^−1^): 3173w, 3010m, 2987m, 2975m, 2910m, 2831m, 2683m, 2583m, 2499m, 1619s, 1544vs, 1502s, 1474m, 1458s, 1423m, 1384vs, 1371vs, 1349s, 1307m, 1274s, 1251s, 1211m, 1192m, 1173s, 1144m, 1108m, 1093s, 1067s, 1017w, 999w, 988w, 972w, 952m, 919w, 888m, 873s, 834m, 803s, 775vvs, 739vs, 640w, 627s, 594s, 552m, 522m, 492m, 475m, 453s.

**X-ray diffraction analysis.** Agilent SuperNova diffractometer (Agilent Technologies XRD Products, Oxfordshire, UK) with molybdenum (Mo-K*_α_*, *λ* = 0.71073 Å) micro-focus sealed X-ray source was used to obtain X-ray diffraction data on single crystal at 150 K. The diffractometer was equipped with mirror optics and an Atlas detector. The crystals were placed on a glass fiber tip with silicon grease, which was mounted on the goniometer head. CrysAlis PRO [27] was used for data processing. Structures were solved with Olex^2^ software [28] using intrinsic phasing in ShelXT [29] and refined with the least squares method in ShelXL [30]. Anisotropic displacement parameters were determined for all non-hydrogen atoms. With the exception of **2b**, NH_3_^+^ and OH hydrogen atoms of protonated amino alcohols were located from a difference Fourier map and refined with isotropic displacement parameters. Owing to the residual density in **2b**, the hydrogen atoms of NH_3_^+^ moiety were added in calculated positions. The residual density, i.e., a 2.30 e^−^/Å^3^ peak on a special position with too-short contacts to adjacent atoms, could not be interpreted. The data set, obtained from a crystal from a different batch, revealed the same problem. The remaining hydrogen atoms were placed in geometrically calculated positions in all structures and refined using riding models. Crystal structure analysis was performed with the program Platon [31], while the figures were made with Mercury [32]. The crystallographic data are summarized in Table 4. All crystal structures were deposited to the Cambridge Crystallographic Data Center (CCDC) and were assigned deposition numbers 2100261 (**1**), 2100262 (**2a**), 2100263 (**2b**), 2100264 (**3a**), and 2100265 (**3b**). These data can be obtained free of charge via http://www.ccdc.cam.ac.uk/conts/retrieving.html (accessed on 15 October 2021) (or from the CCDC, 12 Union Road, Cambridge CB2 1EZ, UK; Fax: +44 1223 336033; E-mail: deposit@ccdc.cam.ac.uk).

## 4. Conclusions

Reactions of amino alcohols (3-amino-1-propanol, 2-amino-1-butanol, or 2-amino-2-methyl-1-propanol) and quinaldinic acid have produced salts, which consist of protonated amino alcohol and deprotonated quinaldinic acid. The obtained products obey the *∆*pK_a_ rule. Of the three products, (3a1pOHH)quin (**1**), (2a1bOHH)quin (**2**), and (2a2m1pOHH)quin (**3**), the last two are polymorphic. A structural survey has revealed all five possible heterosynthons in their crystal structures. The supramolecular structures of all are built of the NH_3_^+^···^−^OOC synthon in combination with one to up to three other heterosynthons. Interestingly, the OH···N(quin^−^) synthon occurs only in one phase. The **2a**/**2b** and **3a**/**3b** polymorphic pairs differ both in the types of hydrogen bonds and in *π*∙∙∙*π* stacking interactions. Due to the former, they are hydrogen bond isomers of the same compound. The presented series is yet another demonstration of polymorphism among molecular solids.

## Figures and Tables

**Table 1:** *π*∙∙∙*π* stacking and C–H∙∙∙*π* interactions [Å, °] in title compounds.

1
***π*∙∙∙*π* Stacking Interactions** Py∙∙∙Py [1−*x*, −*y*, 1−*z*], *Cg*∙∙∙*Cg* = 3.7402(15), dihedral angle = 0.02(11), shift distance = 1.552 Ph∙∙∙Py[1−*x*, −*y*, 1−*z*], *Cg*∙∙∙*Cg* = 3.6571(15), dihedral angle = 0.41(11), shift distance = 1.346 Ph∙∙∙Py[1−*x*, −1−*y*, 1−*z*], *Cg*∙∙∙*Cg* = 3.9681(16), dihedral angle = 0.41(11), shift distance = 1.656 Ph∙∙∙Ph[1−*x*, −1−*y*, 1−*z*], *Cg*∙∙∙*Cg* = 3.6899(16), dihedral angle = 0.00(11), shift distance = 0.821
**2a**
***π*∙∙∙*π* Stacking Interactions** Py∙∙∙Py[−*x*, 1−*y*, 1−*z*], *Cg*∙∙∙*Cg* = 3.7080(10), dihedral angle = 0.02(6), shift distance = 1.635 Ph∙∙∙Py[−*x*, 1−*y*, 1−*z*], *Cg*∙∙∙*Cg* = 3.5163(9), dihedral angle = 0.39(7), shift distance = 1.123
**C–H∙∙∙*π* Interactions** C–H∙∙∙Ph[1−*x*, 1−*y*, 1−*z*], H∙∙∙*Cg* = 3.00, C–H∙∙∙*Cg* = 149, C∙∙∙*Cg* = 3.8620(17)
**2b**
**C–H∙∙∙*π* Interactions** C–H∙∙∙Ph[1+*x*, 1+*y*, *z*], H∙∙∙*Cg* = 2.79, C–H∙∙∙*Cg* = 131, C∙∙∙*Cg* = 3.495(3)
**3a**
**C–H∙∙∙*π* Interactions** C–H∙∙∙Py[2.5−*x*, 0.5+*y*, 0.5−*z*], H∙∙∙*Cg* = 2.95, C–H∙∙∙*Cg* = 150, C∙∙∙*Cg* = 3.7898(19) C–H∙∙∙Ph[1.5−*x*, −0.5+*y*, 0.5−*z*], H∙∙∙*Cg* = 2.78, C–H∙∙∙*Cg* = 140, C∙∙∙*Cg* = 3.5438(18)
**3b**
***π*∙∙∙*π* Stacking Interactions** Ph∙∙∙Py[−*x*, −*y*, 1−*z*], *Cg*∙∙∙*Cg* = 3.8194(7), dihedral angle = 2.51(6), shift distance = 1.462
**C–H∙∙∙*π* Interactions** C–H∙∙∙Py[1+*x*, 1+*y*, *z*], H∙∙∙*Cg* = 2.85, C–H∙∙∙*Cg* = 162, C∙∙∙*Cg* = 3.7689(15) C–H∙∙∙Ph[1+*x*, 1+*y*, *z*], H∙∙∙*Cg* = 2.86, C–H∙∙∙*Cg* = 167, C∙∙∙*Cg* = 3.8079(14)

**Table 2:** Hydrogen bonds (Å) in title compounds.

Compound	Synthon	Details
**1**	NH_3_^+^∙∙∙^−^OOC	N∙∙∙O[2−*x*, 1−*y*, 2−*z*] = 2.740(3)
	NH_3_^+^∙∙∙^−^OOC	N∙∙∙O[*x*, 1+*y*, *z*] = 2.817(3)
	OH∙∙∙^−^OOC	O∙∙∙O = 2.739(3)
**2a**	NH_3_^+^∙∙∙^−^OOC	N∙∙∙O = 2.8147(14)
	NH_3_^+^∙∙∙^−^OOC	N∙∙∙O[0.5+*x*, 0.5−*y*, 0.5+*z*] = 2.8216(16)
	NH_3_^+^∙∙∙OH	N∙∙∙O[1−*x*, −*y*, 1−*z*] = 2.9409(14)
	OH∙∙∙^−^OOC	O∙∙∙O = 2.6178(12)
**2b**	NH_3_^+^∙∙∙^−^OOC	N∙∙∙O = 2.772(3)
	NH_3_^+^∙∙∙^−^OOC	N∙∙∙O[1+*x*, *y*, *z*] = 2.948(2)
	NH_3_^+^∙∙∙^−^OOC	N∙∙∙O[1−*x*, 1−*y*, 1−*z*] = 3.008(2)
	NH_3_^+^∙∙∙N(quin^−^)	N∙∙∙N[1+*x*, *y*, *z*] = 3.080(3)
	OH∙∙∙^−^OOC	O∙∙∙O = 2.791(2)
**3a**	NH_3_^+^∙∙∙^−^OOC	N∙∙∙O = 2.7404(18)
	NH_3_^+^∙∙∙^−^OOC	N∙∙∙O[−1+*x*, *y*, *z*] = 2.7768(18)
	NH_3_^+^∙∙∙^−^OOC	N∙∙∙O[1−*x*, 1−*y*, 1−*z*] = 2.7976(16)
	OH∙∙∙N(quin^−^)	O∙∙∙N[−1+*x*, *y*, *z*] = 2.8187(17)
**3b**	NH_3_^+^∙∙∙^−^OOC	N∙∙∙O[1−*x*, 2−*y*, 2−*z*] = 2.7307(13)
	NH_3_^+^∙∙∙^−^OOC	N∙∙∙O[*x*, 1+*y*, *z*] = 2.8525(13)
	NH_3_^+^∙∙∙OH	N∙∙∙O[1−*x*, 2−*y*, 2−*z*] = 2.8148(12)
	OH∙∙∙^−^OOC	O∙∙∙O = 2.6222(12)

**Table 3:** Heterosynthon occurrence in the structures of title compounds.

	1	2a	2b	3a	3b
NH_3_^+^∙∙∙^−^OOC	✓	✓	✓	✓	✓
OH∙∙∙^−^OOC	✓	✓	✓		✓
NH_3_^+^∙∙∙N(quin^−^)	✓ ^[a]^	✓ ^[a]^	✓		✓ ^[a]^
NH_3_^+^∙∙∙OH		✓			✓
OH∙∙∙N(quin^−^)				✓	

^[a]^ Weak interaction. The N∙∙∙N contact is longer than the sum of the corresponding van der Waals radii, 3.1 Å [17].

**Table 4:** Crystallographic data for **1**–**3b**.

	1	2a	2b	3a	3b
**Empirical Formula**	C_13_H_16_N_2_O_3_	C_14_H_18_N_2_O_3_	C_14_H_18_N_2_O_3_	C_14_H_18_N_2_O_3_	C_14_H_18_N_2_O_3_
**Formula Weight**	248.28	262.30	262.30	262.30	262.30
**Crystal System**	triclinic	monoclinic	monoclinic	monoclinic	triclinic
**Space Group**	*P*−1	*P* 2_1_/*n*	*P* 2_1_/*n*	*P* 2_1_/*n*	*P*−1
***T* (K)**	150.00(10)	150.00(10)	150.00(10)	150.00(10)	150.00(10)
***λ* (Å)**	0.71073	0.71073	0.71073	0.71073	0.71073
***a* (Å)**	7.1378(16)	12.1437(11)	6.5579(4)	6.5428(4)	7.1342(4)
***b* (Å)**	7.5269(7)	10.1451(5)	10.2309(6)	9.0723(4)	8.4346(3)
***c* (Å)**	11.8314(14)	12.2312(15)	19.8329(13)	23.1232(10)	12.5059(7)
***α* (°)**	99.172(9)	90	90	90	96.139(4)
***β* (°)**	95.916(14)	119.527(14)	97.837(6)	93.835(5)	105.187(5)
***γ* (°)**	90.647(13)	90	90	90	104.829(4)
***V* (Å^3^)**	623.93(17)	1311.2(3)	1318.22(14)	1369.48(12)	689.74(6)
***Z***	2	4	4	4	2
***D*_calc_ (g/cm^3^)**	1.322	1.329	1.322	1.272	1.263
**μ (mm^−1^)**	0.095	0.094	0.094	0.090	0.090
**Collected Reflections**	5349	11,880	6877	12,825	12,006
**Unique Reflections**	3186	3524	3413	3696	3689
**Observed Reflections**	1937	2780	2459	2528	2933
***R*_int_**	0.0587	0.0285	0.0233	0.0482	0.0224
***R*_1_ (*I* > 2*σ*(*I*))**	0.0878	0.0413	0.0671	0.0513	0.0429
***wR*_2_ (all data)**	0.2559	0.1168	0.2000	0.1225	0.1274

## Supplementary Materials

The following supporting information can be downloaded, PXRD patterns (Figures S1–S3), packing diagrams (Figures S4–S6), IR spectra for 1–3b (Figures S7–S11), 1H NMR spectra for 1–3 (Figures S12–S14), and TG/DSC curves (Figures S15–S17).

## Sample Availability

Obtained from the authors upon request.
