# Supplementary material for: Hydrogen Bonding and Polymorphism of Amino Alcohol Salts with Quinaldinate: Structural Study
Source: Molecules. 2022 Feb 1;27(3):996. doi: 10.3390/molecules27030996 (PMC8839704; doi:10.3390/molecules27030996)

Supplementary materials for

Hydrogen bonding and polymorphism of amino alcohol salts with quinaldinate: Structural study

Nina Podjed, Barbara Modec*

Faculty of Chemistry and Chemical Technology, University of Ljubljana, Večna pot 113
1000 Ljubljana, Slovenia

* Corresponding author. E-mail: barbara.modec@fkkt.uni-lj.si

Abbreviations

**1**	(3a1pOHH)quin
**2**	(2a1bOHH)quin
**3**	(2a2m1pOHH)quin
quin^{-}	anion of quinaldinic acid
3a1pOHH^{+}	protonated 3-amino-1-propanol
2a1bOHH^{+}	protonated 2-amino-1-butanol
2a2m1pOHH^{+}	protonated 2-amino-2-methyl-1-propanol

2a and **2b** are polymorphs of **2**, **3a** and **3b** are polymorphs of **3**.

1. Powder X-ray diffraction patterns

Figure S1. Comparison of the calculated powder pattern for **1** with the measured PXRD pattern of the product of the direct reaction between 3-amino-1-propanol and quinaldinic acid.

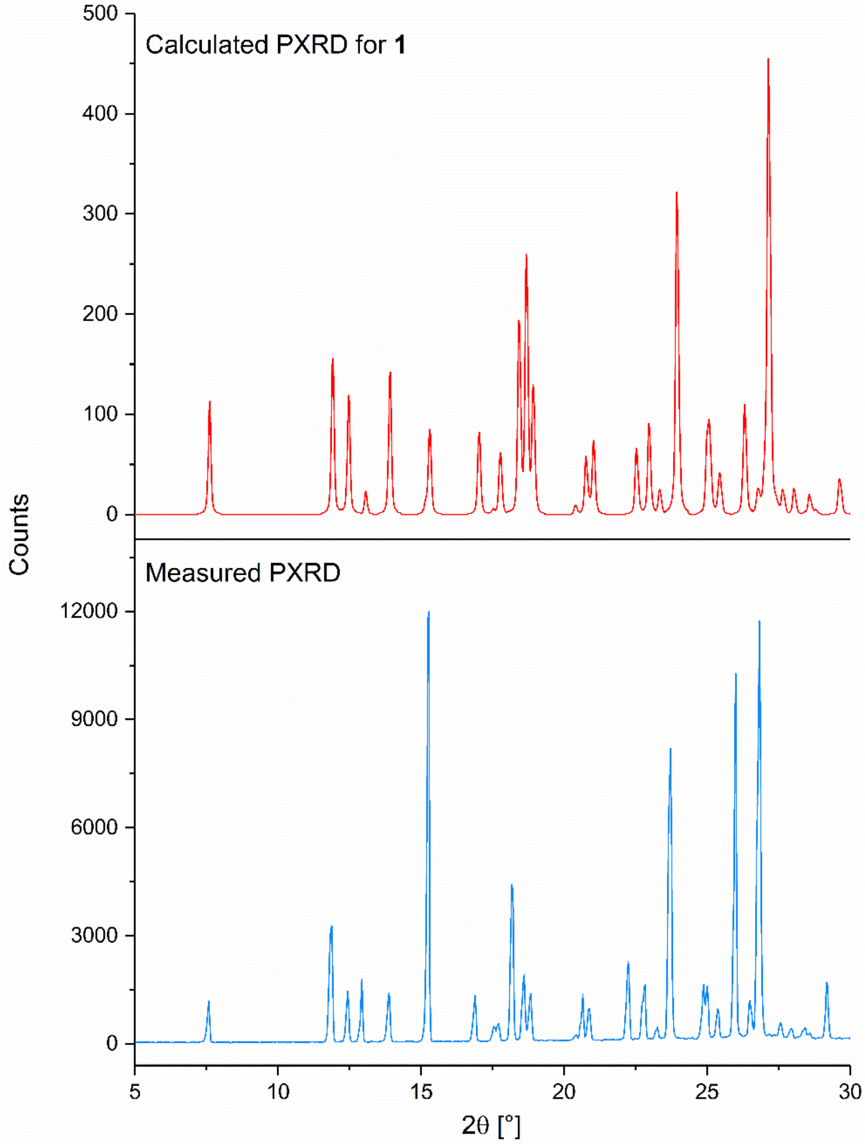

Figure S2. Comparison of the calculated powder patterns for **2a** and **2b** polymorphs with the measured PXRD pattern of the product of the direct reaction between 2-amino-1-butanol and quinaldinic acid.

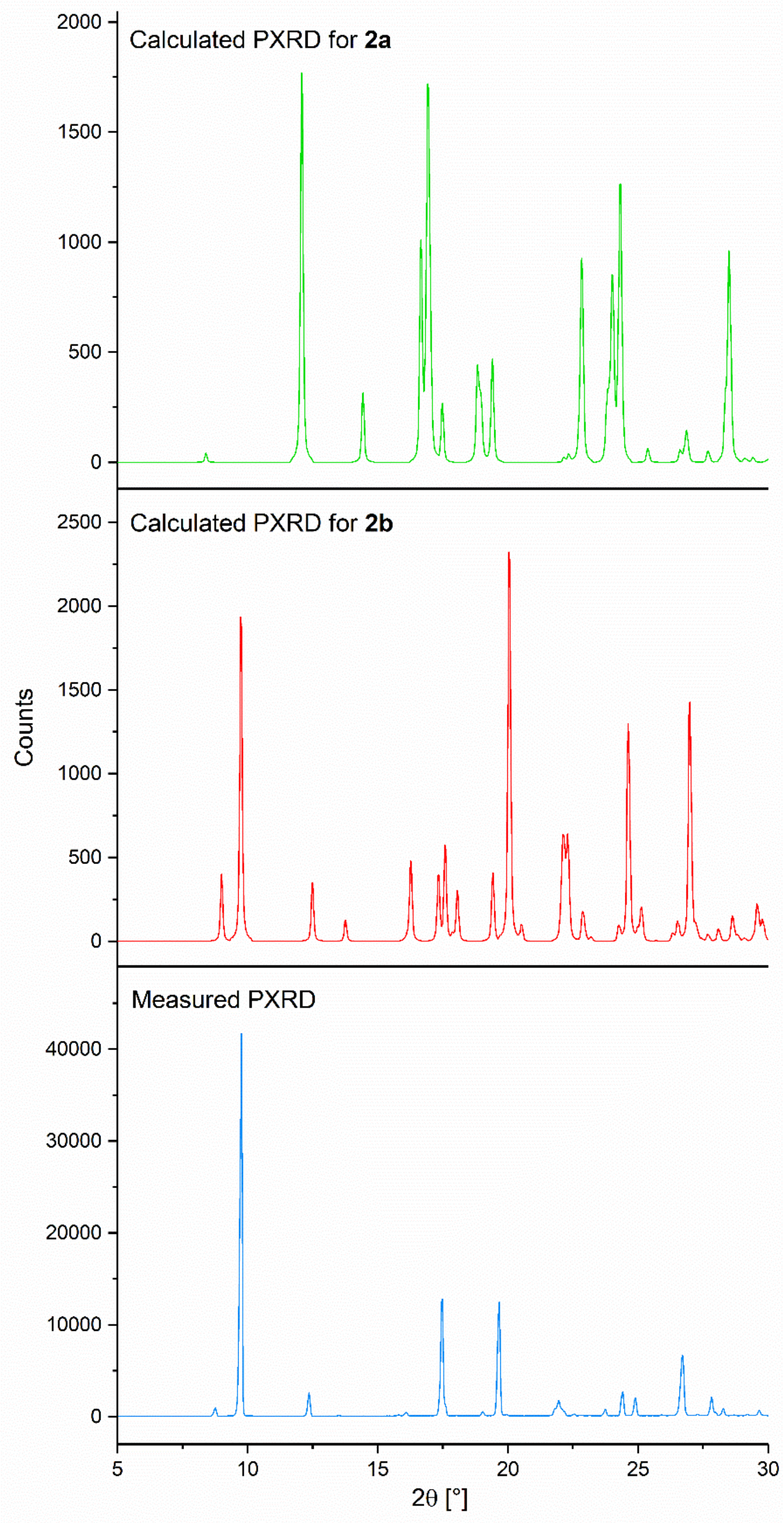

Figure S3. Comparison of the calculated powder patterns for **3a** and **3b** polymorphs with the measured PXRD pattern of the product of the direct reaction between 2-amino-2-methyl-1-propanol and quinaldinic acid.

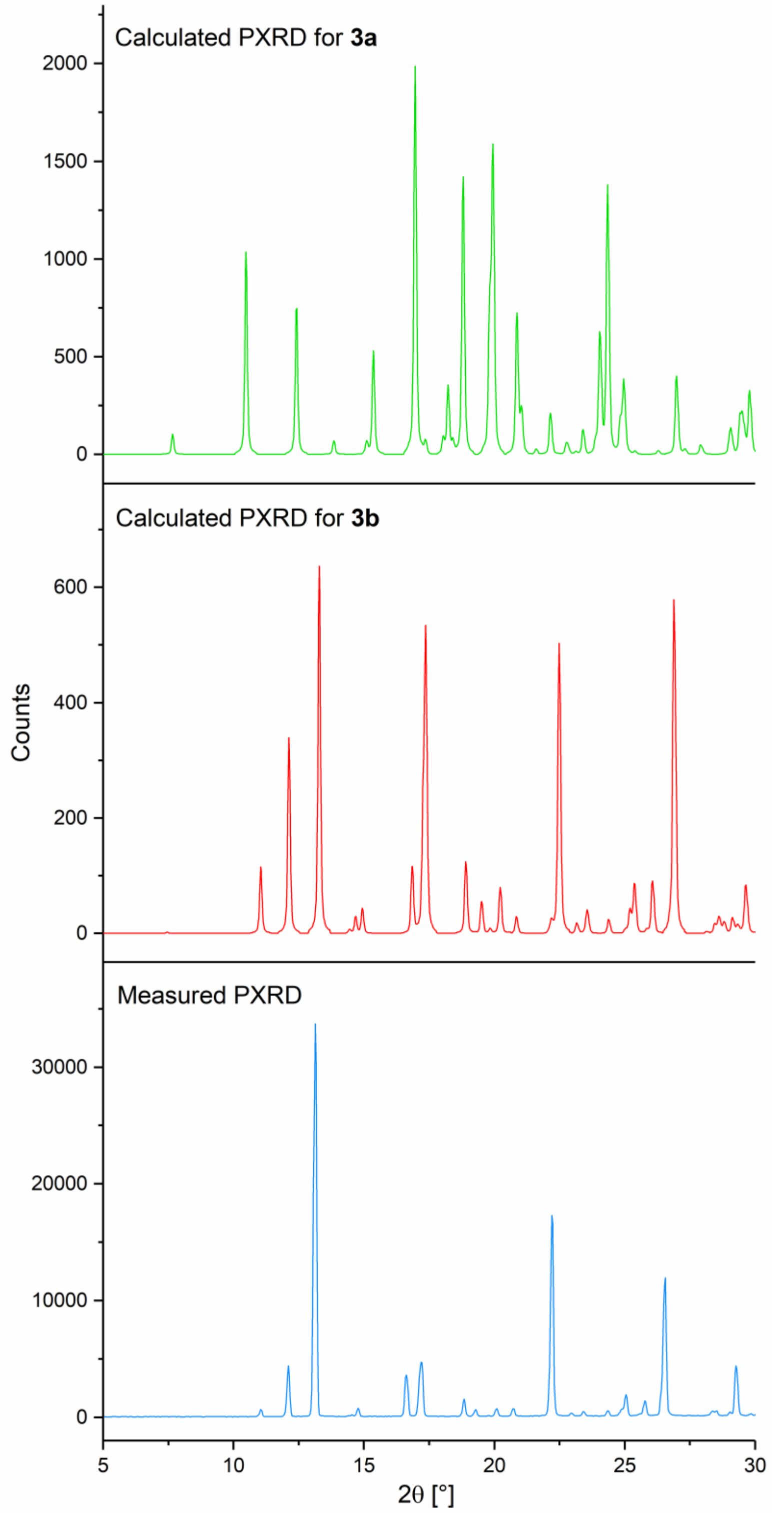

2. Packing diagrams

Figure S4. Stacking of layers in **1**: a view along one (top) or two (bottom) layers. From these two projections, $\pi\cdots\pi$ stacking interactions occurring between quinaldinates from adjacent layers may be seen.

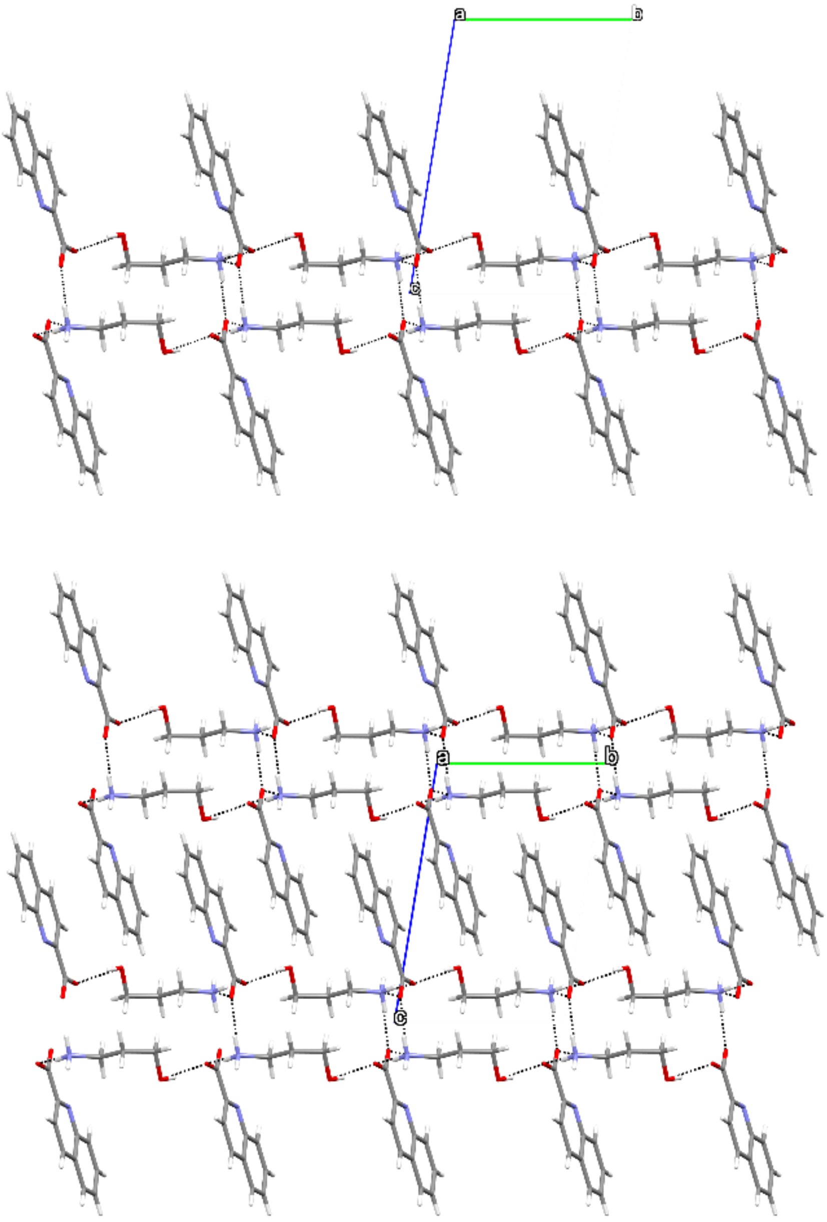

Figure S5. A view along one layer (top) or two layers (bottom) in **2a**. From these two projections, $\pi\cdots\pi$ stacking interactions occurring between quinaldinates from adjacent layers may be seen.

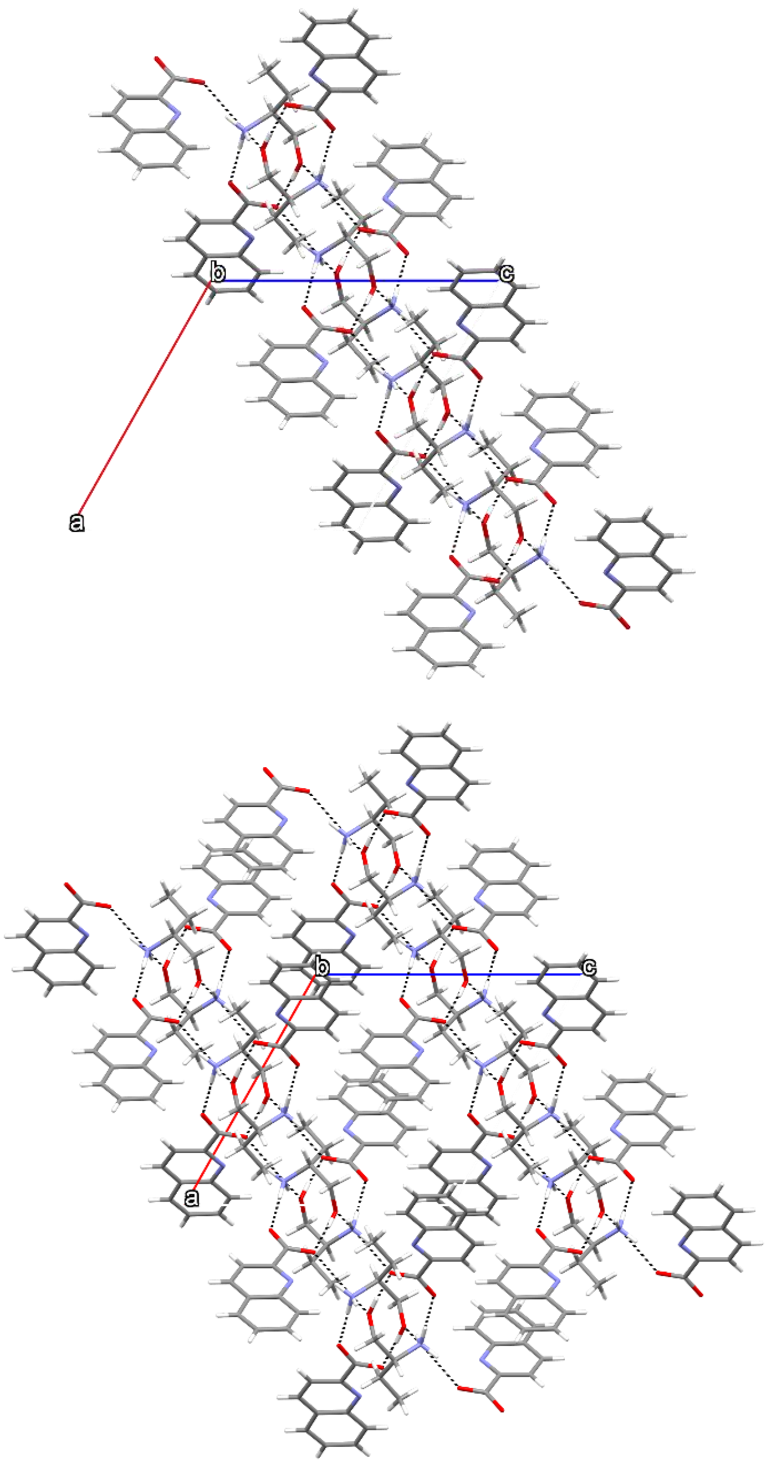

Figure S6. A view along one (top) or three (bottom) chains in the **3b** polymorph. From these two projections, $\pi \cdots \pi$ stacking interactions occurring between quinaldinates from adjacent chains may be seen.

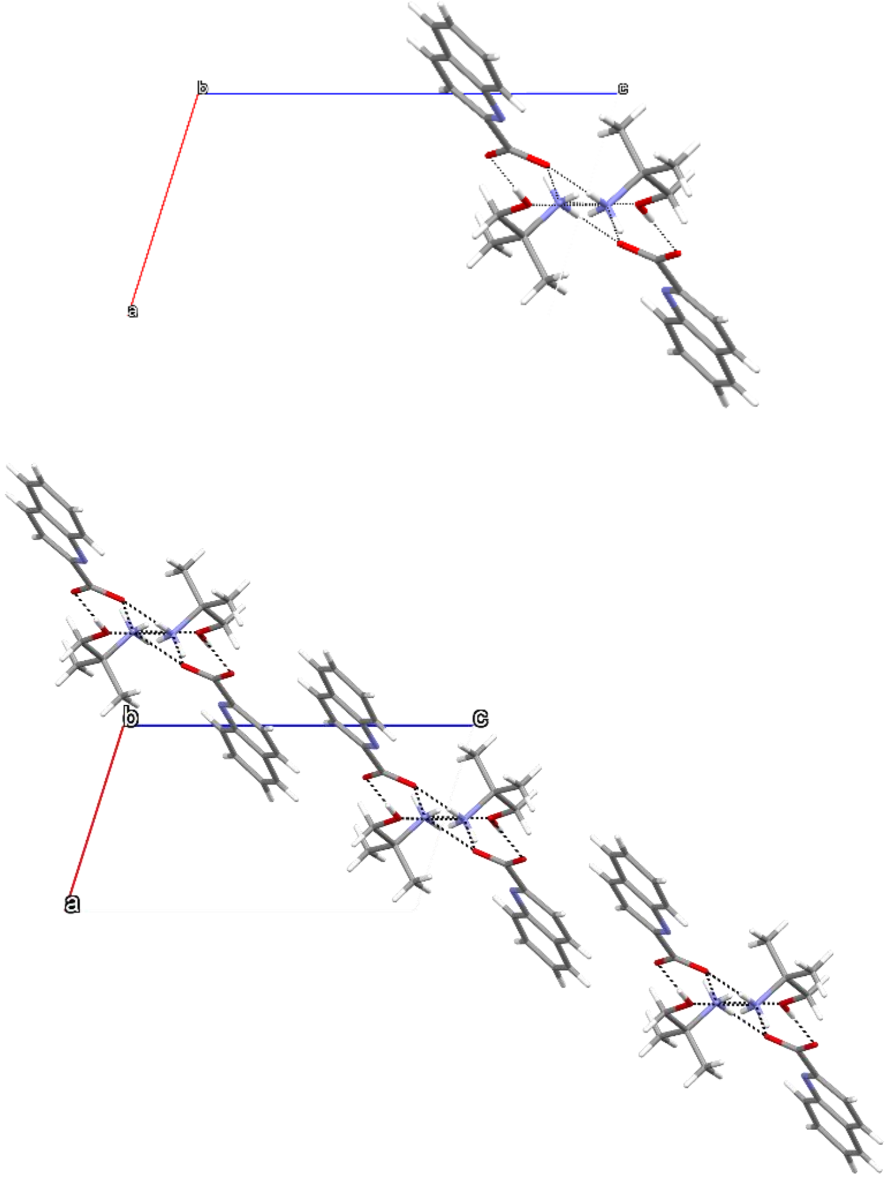

3. IR spectra

Figure S7. IR spectrum of **1**.

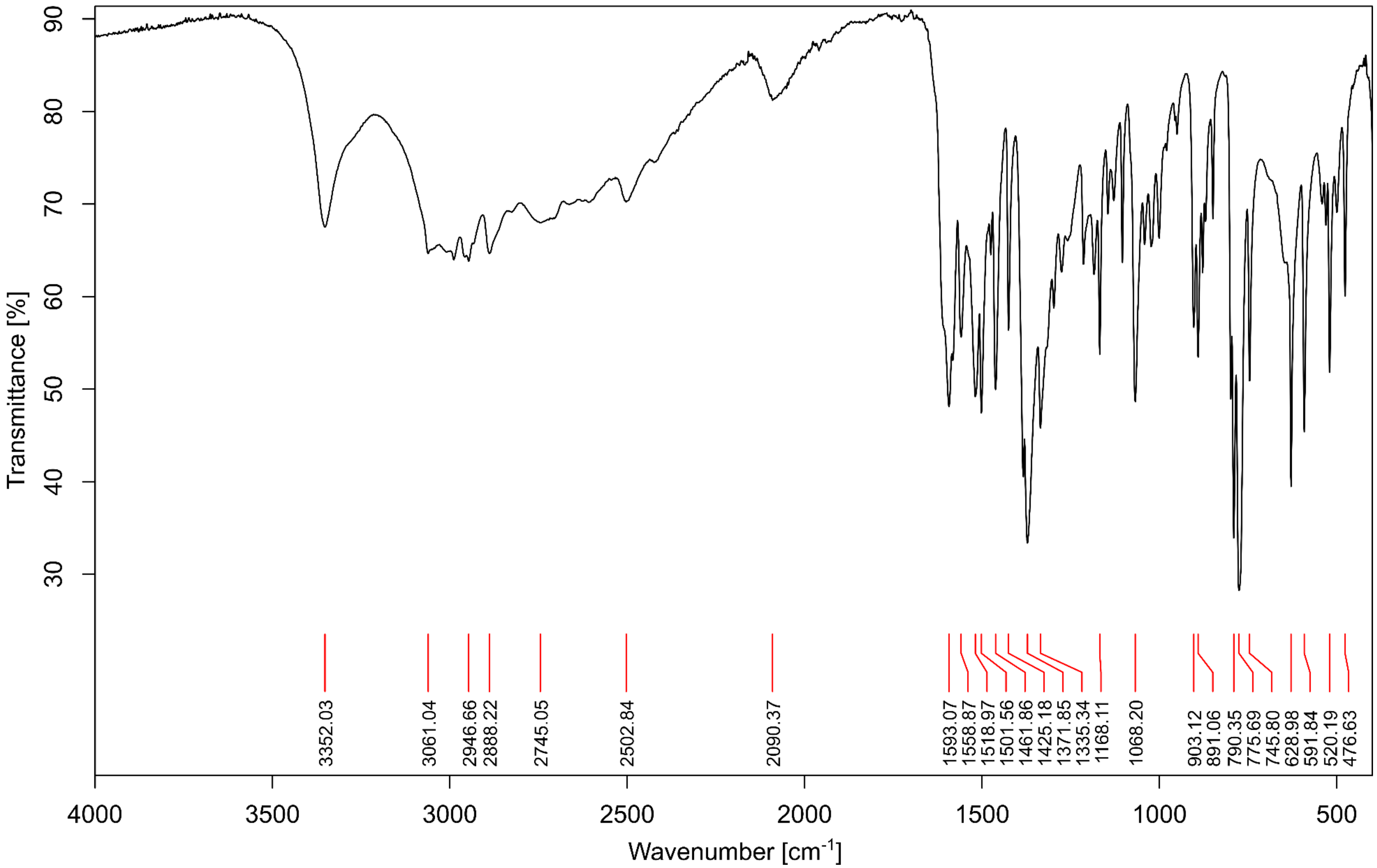

Figure S8. IR spectrum of the **2a** polymorph.

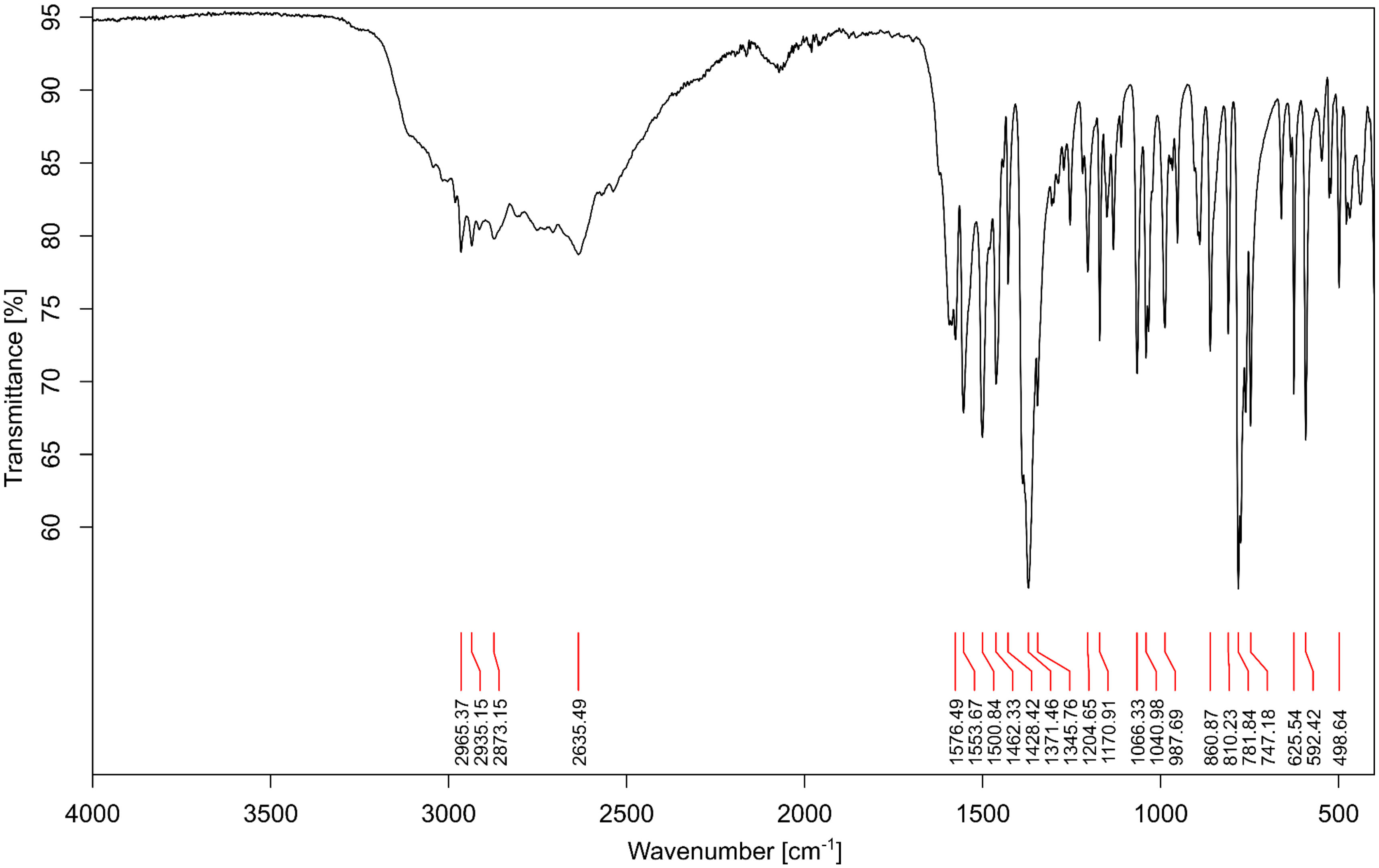

Figure S9. IR spectrum of the **2b** polymorph.

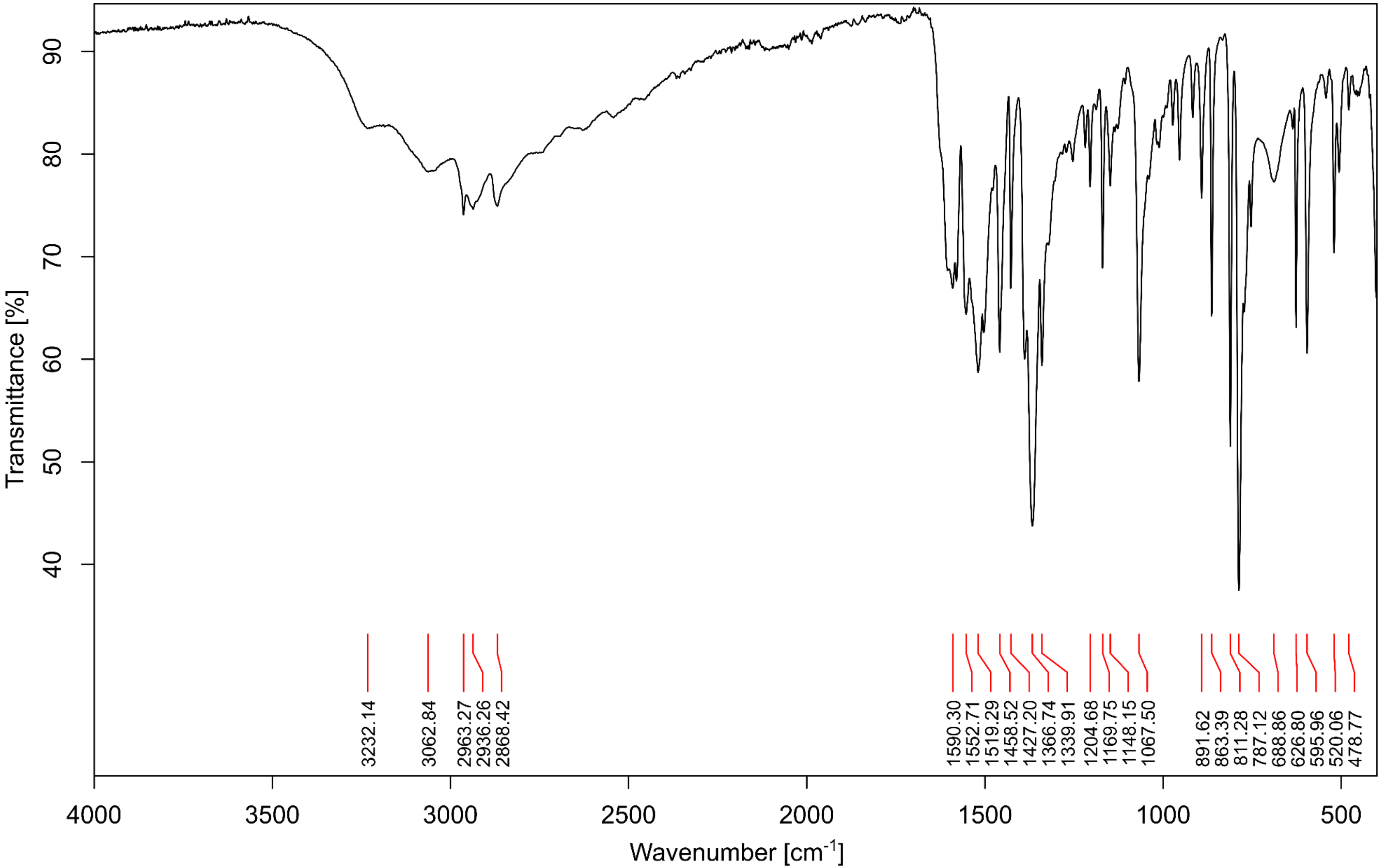

Figure S10. IR spectrum of the **3a** polymorph.

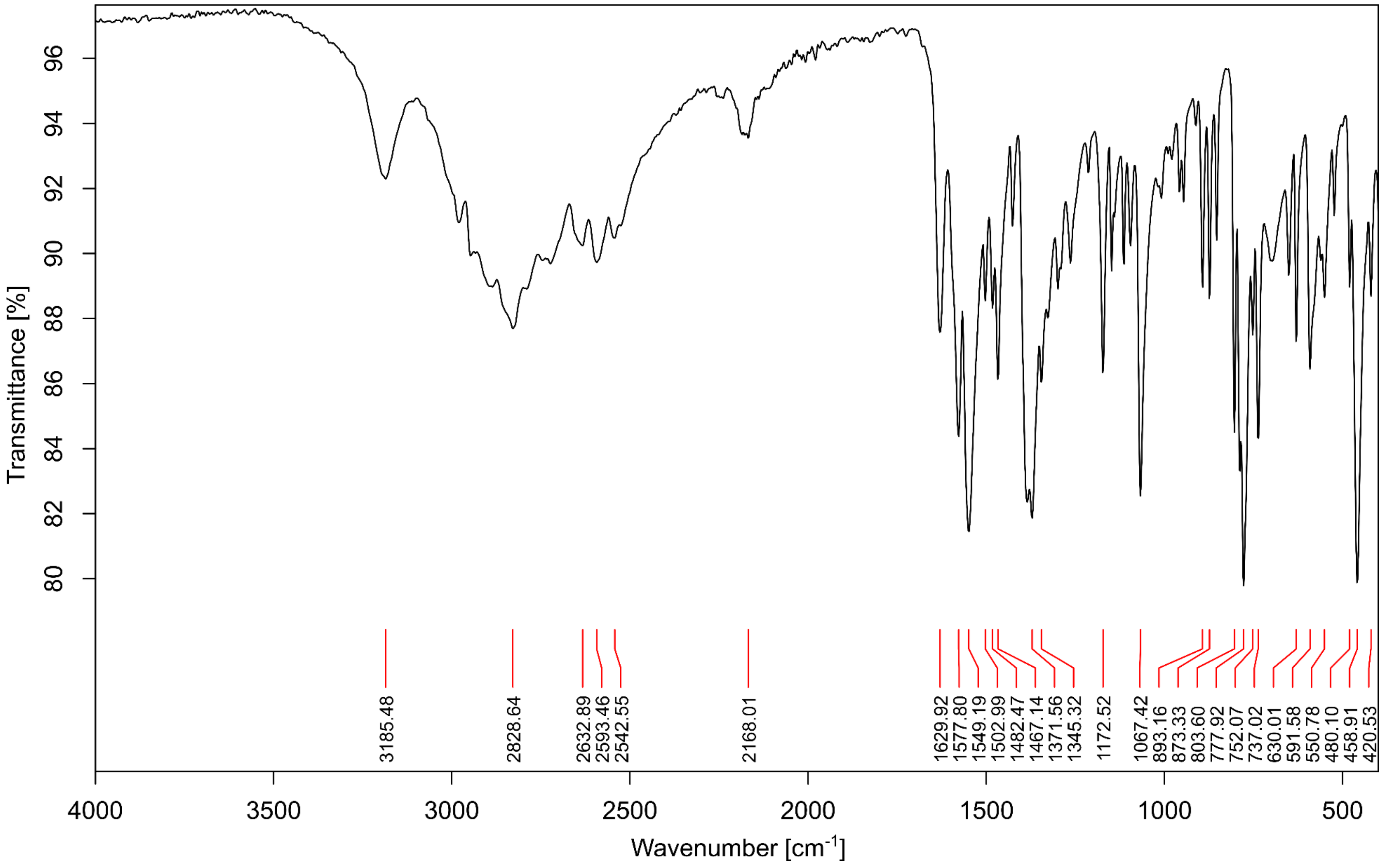

Figure S11. IR spectrum of the **3b** polymorph.

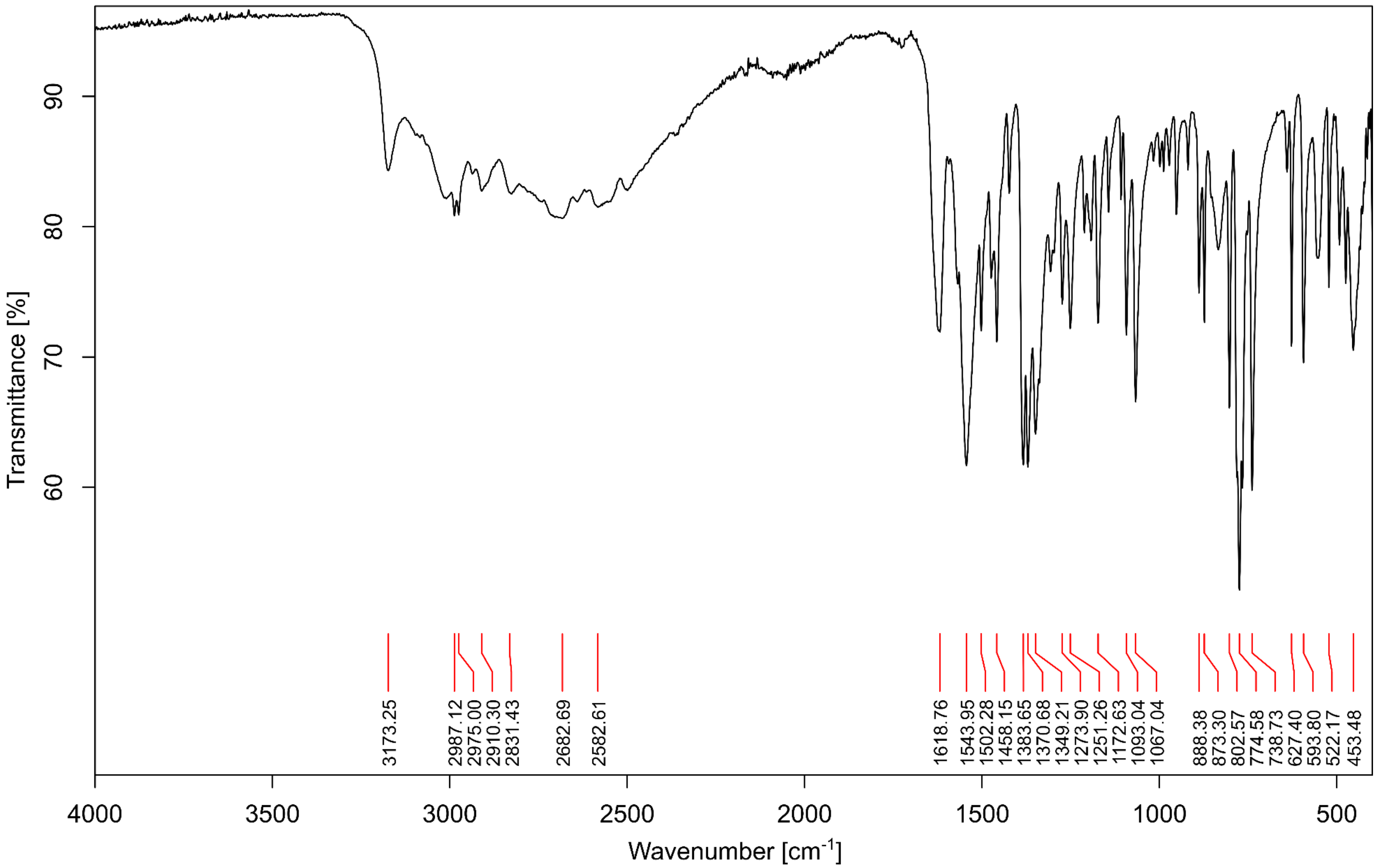

4. ^{1}H NMR spectra

Figure S12. ^{1}H NMR spectrum of **1** in DMSO-d_6.

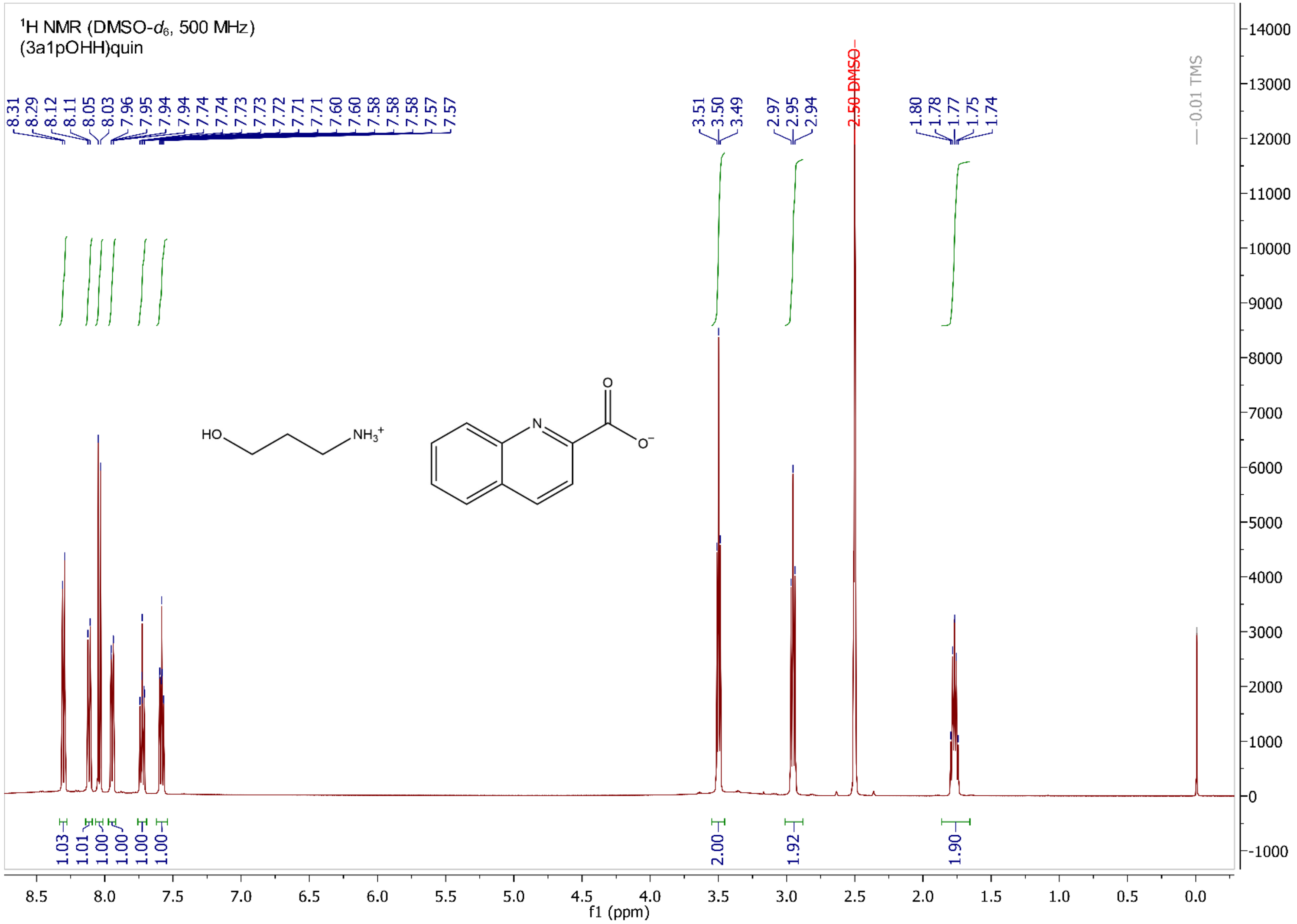

Figure S13. ^{1}H NMR spectrum of **2** in DMSO-d_6.

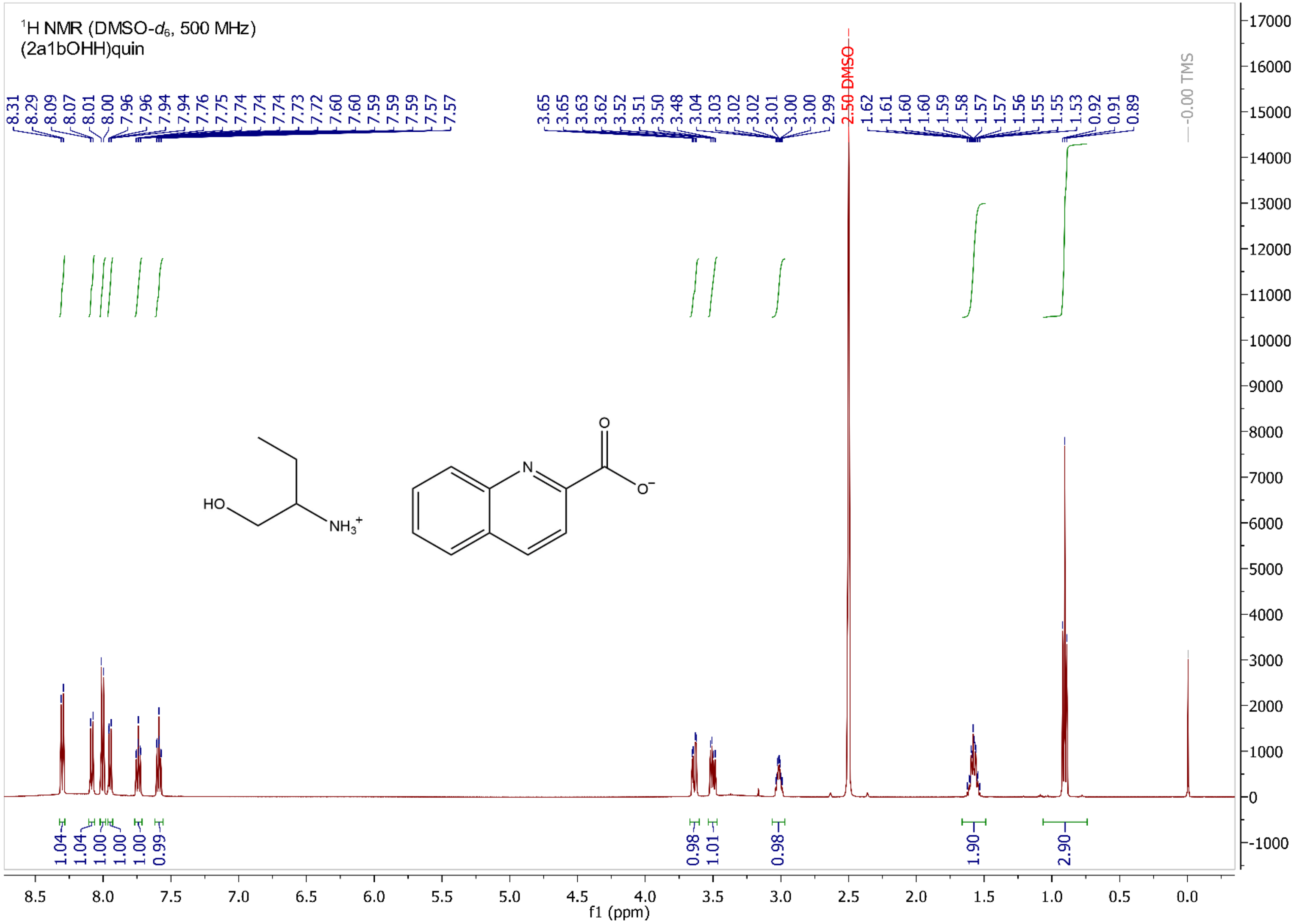

Figure S14. ^1H NMR spectrum of **3** in DMSO-d_6.

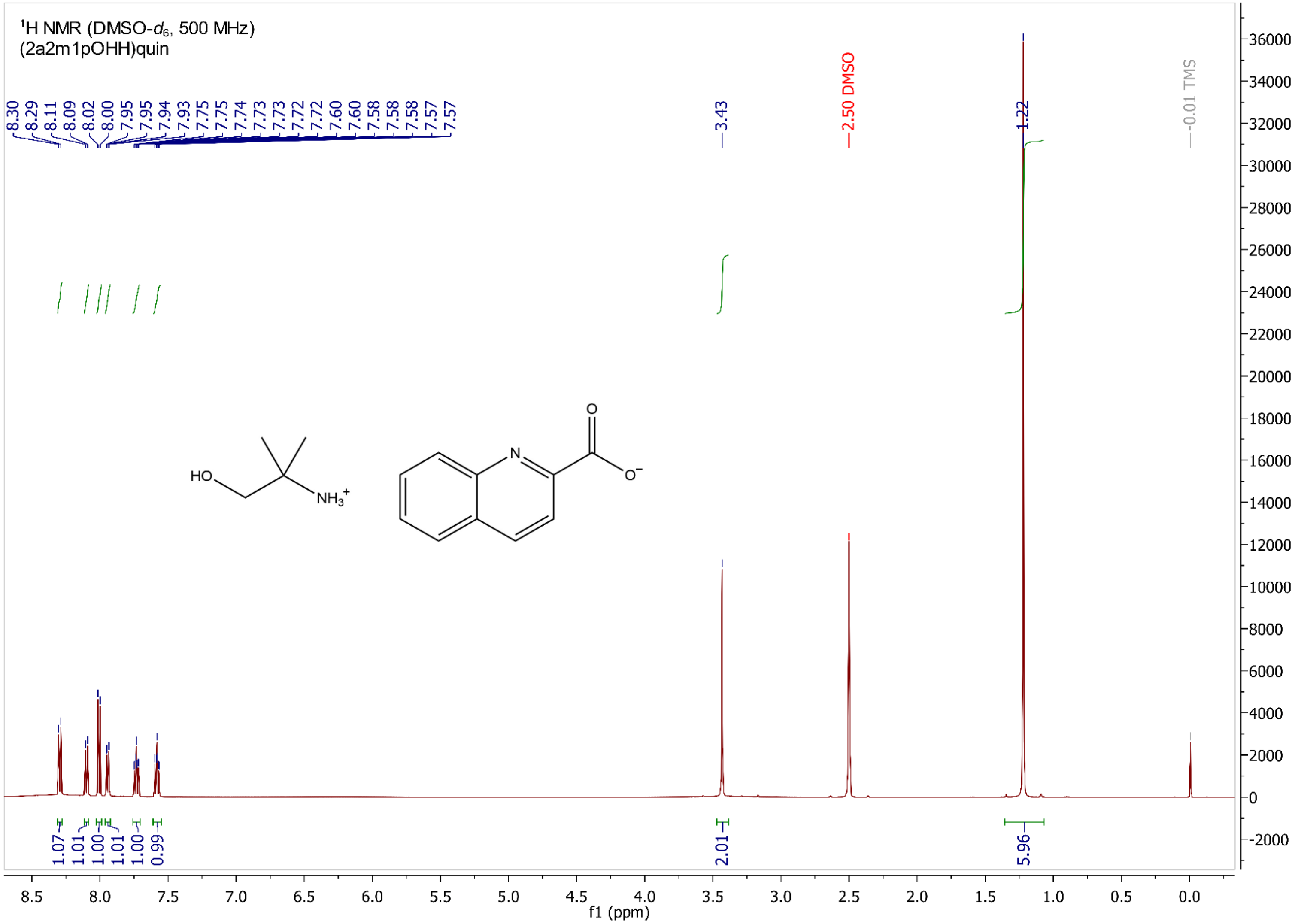

5. TG/DSC curves.

Figure S15. TG and DSC curves for **1**.

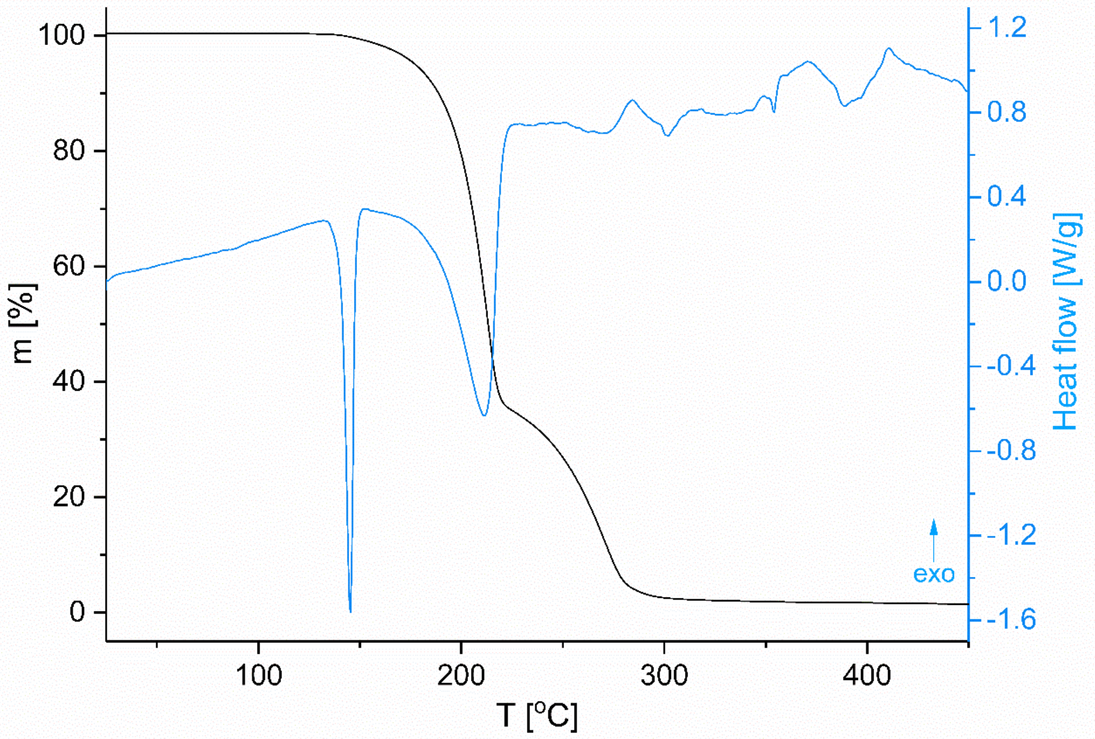

Figure S16. TG and DSC curves for **2b**.

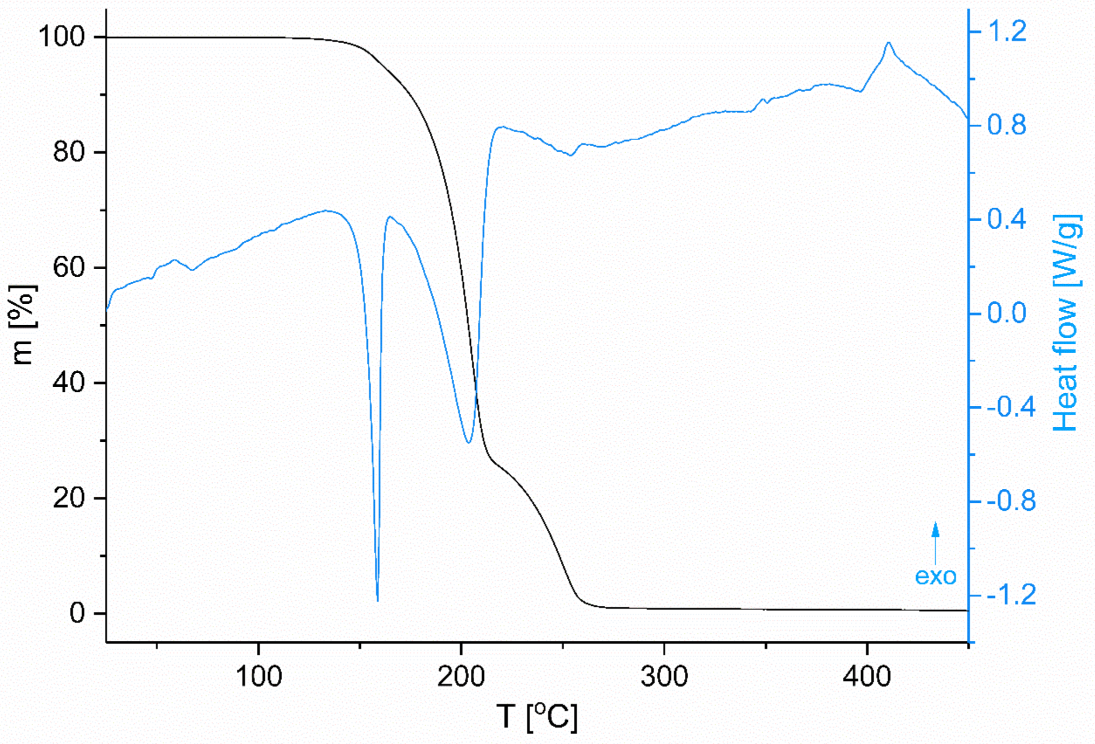

Figure S17. TG and DSC curves for **3b**.

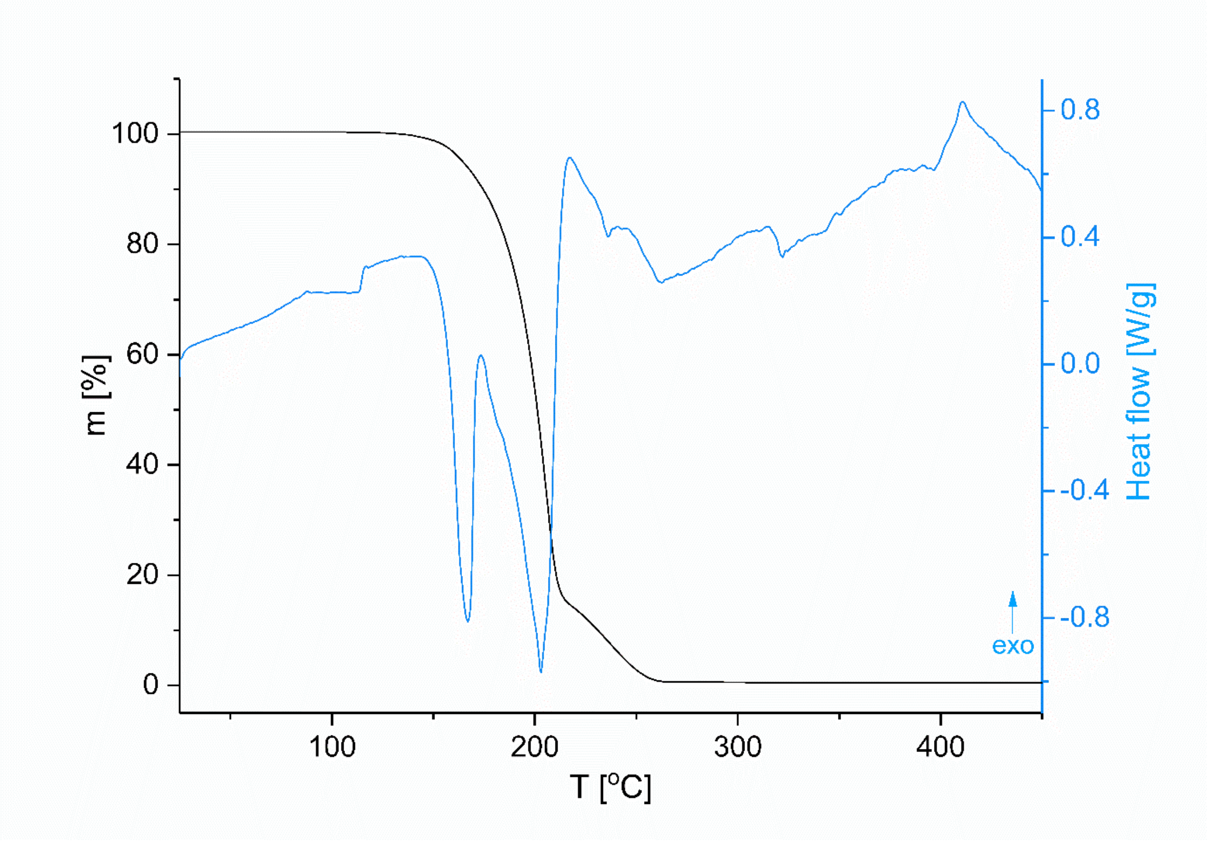

Supplement: Supplementary file 1 [file molecules-27-00996-s001.zip › molecules-1542321-supplementary.pdf]
